# Evaluation of Potential Anti-Diabetic Synbiotic Formulation of *Lacticaseibacillus rhamnosus* BST.L-601 Using db/db Mice

**DOI:** 10.3390/foods14244230

**Published:** 2025-12-09

**Authors:** Hyewon Choe, Chae Young Shin, Jung Sik Lim, Jung-Soo Park, Joo-Woong Park, Woo Jung Kim, Yong Il Park, Jin Ree

**Affiliations:** 1 Biostream Co., Ltd., Suwon 10442, Republic of Korea; hwchoe@biostream.co.kr (H.C.); jspark@biostream.co.kr (J.-S.P.); pjwrnds@biostream.co.kr (J.-W.P.); 2 Department of Biotechnology, Graduate School, The Catholic University of Korea, Bucheon 14662, Republic of Korea; 4758shyg@daum.net (C.Y.S.); ti5759@naver.com (J.S.L.); 3 Biocenter, Gyeonggi-do Business and Science Accelerator, Suwon 16229, Republic of Korea; wj0504@gbsa.co.kr; 4 Department of Medical and Biological Sciences, The Catholic University of Korea, Bucheon 14662, Republic of Korea

**Keywords:** *Lacticaseibacillus rhamnosus*, probiotics, symbiotics, type 2 diabetes mellitus, microbiome diversity, probiotics properties

## Abstract

Probiotics have been studied for their potential to treat chronic diseases. This study examined the use of *Lacticaseibacillus rhamnosus* BST.L-601 as an anti-diabetic symbiotic with sweet potato for fermentation. The medium supplemented with sweet potato showed increased productivity and enhanced storability. The anti-diabetic effect of fermented BST.L-601 was evaluated using the C2C12 myotube and a type 2 diabetes mellitus (T2DM)-induced db/db (Lepr^db^/Lepr^db^) mouse model. Treatment with heat-killed BST.L-601 increased glucose uptake by 125% and α-glucosidase inhibition in a dose-dependent manner without cytotoxicity for myotubes. 8 weeks of oral administration of BST.L-601 led to anti-diabetic activities in various biomarkers in the mouse model, including lowered fasting blood glucose by 88% and elevated mRNA expression of glucose metabolism-related factors IRS-1 (510%) and GLUT4 (181%) from skeletal muscle. Moreover, the improvement of induced T2DM in mice was supported by blood serum analysis. Immunohistochemistry showed increased insulin and decreased glucagon secreted from β and α cells in the pancreas islet. Microbiota analysis demonstrated elevated microbiome diversity in mice treated with BST.L-601. Furthermore, the safety and probiotic properties of the strain were confirmed. These results suggest that BST.L-601 fermented with sweet potato could be a functional symbiotic used to improve diabetes, particularly T2DM.

## 1. Introduction

Diabetes mellitus (DM) is a globally prevalent metabolic disorder characterized by chronic hyperglycemia resulting from impaired insulin secretion, insulin resistance, or both. Type 2 diabetes mellitus (T2DM), which accounts for more than 90% of DM cases, is strongly associated with insulin resistance and β-cell dysfunction, predisposing patients to micro- and macrovascular complications, including retinopathy, nephropathy, neuropathy, and cardiovascular diseases. The World Health Organization estimates that diabetes will become one of the leading causes of global morbidity and mortality within the next decade, underscoring the urgent need for effective prevention and management strategies [1,2]. Despite the availability of several pharmacological therapies, the adverse effects and long-term limitations of these agents highlight the need for safer and more sustainable interventions [3].

Conventional anti-diabetic drugs, including insulin, metformin, sulfonylureas, and sodium–glucose cotransporter 2 (SGLT2) inhibitors, play pivotal roles in clinical management. However, they are often associated with undesirable side effects, such as hypoglycemia, gastrointestinal disturbances, weight gain, and increased cardiovascular risk [4,5,6]. In addition, their inability to address the multifactorial nature of T2DM, which involves not only glucose dysregulation but also lipid imbalance, inflammation, and gut microbial dysbiosis, limits their overall efficacy. Consequently, attention has increasingly shifted toward dietary and microbiota-modulating interventions, which may offer complementary or alternative approaches to conventional pharmacotherapy [7,8].

Among these approaches, probiotics—particularly strains of *Lacticaseibacillus rhamnosus*—have emerged as promising candidates for metabolic health modulation. Several clinical and preclinical studies have demonstrated their capacity to improve glucose homeostasis, reduce insulin resistance, modulate lipid metabolism, and restore gut microbial balance [9,10,11]. For example, supplementation with *L. rhamnosus* LRa05 for 12 weeks significantly reduced fasting blood glucose and improved HDL cholesterol levels in patients with T2DM, accompanied by favorable alterations in gut microbiota composition, including increased *Bifidobacterium* and decreased *Firmicutes* populations [10]. Similarly, in db/db (Lepr^db^/Lepr^db^) mice, administration of *L. rhamnosus* Hao9 reduced fasting glucose and insulin concentrations, enhanced hepatic antioxidant defenses, suppressed gluconeogenic enzymes (G6Pase and PEPCK), downregulated pro-inflammatory cytokines, and improved intestinal barrier integrity [11]. These findings indicate that probiotics have a multifaceted role in mitigating metabolic disturbances beyond glycemic control.

Prebiotics, such as dietary fibers and resistant starches, further potentiate the effects of probiotics by selectively stimulating their growth and metabolic activity. Sweet potato (*Ipomoea batatas*) powder, which is rich in resistant starch, polyphenols, and dietary fibers, has been reported to exert anti-diabetic, antioxidant, and anti-inflammatory properties [12,13,14,15]. In animal and human studies, sweet potato supplementation attenuated postprandial glucose excursions, inhibited α-glucosidase activity, and promoted the production of short-chain fatty acids (SCFAs), thereby contributing to improved insulin sensitivity and gut health [12,13,14,15]. Recent research has also demonstrated that resistant starch from sweet potato enhances microbial diversity, particularly increasing SCFA-producing bacteria, which are known to regulate host metabolism and inflammation. Despite these promising findings, the combined use of probiotics and sweet potato as a synbiotic strategy remains underexplored in the context of T2DM.

Synbiotics—defined as the synergistic combination of probiotics and prebiotics—represent an emerging strategy to maximize the health benefits of both components. By providing both live beneficial microorganisms and substrates that enhance their growth, synbiotics offer the potential to modulate host metabolism and immunity more effectively than probiotics or prebiotics alone. Recent studies have shown that synbiotic supplementation can improve glycemic control, lipid metabolism, and inflammatory markers in patients with metabolic syndrome and T2DM [16]. Nevertheless, specific evidence regarding *Lacticaseibacillus rhamnosus* combined with sweet potato powder remains scarce, particularly in well-established T2DM animal models, such as db/db mice.

In addition to efficacy, the stability and safety of probiotic strains are critical for their application as functional food ingredients. Recent investigations emphasize the importance of confirming non-hemolytic activity, absence of cytotoxicity, and susceptibility to clinically relevant antibiotics to ensure consumer safety [17,18]. Furthermore, strategies to improve probiotic viability during fermentation and storage have gained attention, as strain stability is often compromised under industrial processing conditions. Notably, sweet potato powder not only serves as a prebiotic substrate but may also enhance the survival and metabolic resilience of probiotics during cultivation, thereby reinforcing its dual role in synbiotic formulations [19].

We aimed to explore whether the combination of *Lacticaseibacillus rhamnosus* BST.L-601 and sweet potato powder could exhibit potential anti-diabetic effects by improving glucose metabolism, alleviating insulin resistance, and modulating gut microbiota in vitro and in vivo. We further assumed that the insoluble starch components of sweet potato powder might contribute to the stability and viability of the bacterial strain during cultivation and storage, thereby supporting its potential as a safe candidate for functional foods [20,21]. Based on prior research revealing anti-obesity effects of the strain BST.L-601 and its fermented product with sweet potato powder [22], it was anticipated that it would also demonstrate positive efficacy for diabetes, a disorder closely related to obesity from a metabolic perspective. In addition, we postulated that the insoluble starch components of sweet potato powder could enhance the stability and viability of the strain during cultivation and storage, thereby reinforcing its potential as a safe and effective functional food ingredient. Based on this hypothesis, the present study investigated the anti-diabetic efficacy of *L. rhamnosus* BST.L-601 cultured in sweet-potato-powder-supplemented medium using C2C12 myoblasts and db/db mouse models. Particular emphasis was placed on its effects on glucose metabolism, insulin sensitivity, and gut microbiota diversity. To further substantiate its applicability as a synbiotic dietary intervention for T2DM management, we also assessed its safety and stability, including hemolysis, cytotoxicity, and antibiotic susceptibility assays.

## 2. Materials and Methods

### 2.1. Identification of Strain BST.L-601

BST.L-601 was isolated from human stool, and the strain was identified as *Lacticaseibacillus rhamnosus*. Microbial identification of *Lacticaseibacillus rhamnosus* BST.L-601 was conducted, with primer sets of 27F and 1492R, using 16S rRNA gene sequencing (Solgent, Daejeon, Republic of Korea), and the strain was taxonomically classified based on NCBI/BLAST analysis and phylogenetic tree construction [23].

### 2.2. Safety Assessment

The safety of BST.L-601 was confirmed using established methods, with modifications [24,25].

To test the antibiotic susceptibility of BST.L-601, the minimal inhibitory concentration (MIC) of strain BST.L-601 and *Lacticaseibacillus rhamnosus* GG was evaluated using 9 antibiotics: ampicillin, chloramphenicol, clindamycin, erythromycin, gentamycin, kanamycin, streptomycin, tetracycline, and vancomycin. The result was compared to European Food Safety Authority (EFSA) standards [26]. Genetic analysis to identify virulence genes of BST.L-601 was conducted with whole genome sequencing (WGS) based on the VirulenceFinder 2.0. database.

To test the hemolytic activity of BST.L-601, a hemolytic activity assay was conducted. BST.L-601, *L. rhamnosus* GG, *Bacillus* spp., and *E. coli* were streaked on blood agar containing 5% (*w*/*v*) sheep blood and then incubated for 48 h at 37 °C under anaerobic conditions. Hemolytic activity of each strain was classified as alpha (α), beta (β), or gamma (γ) hemolytic according to the colors of hemolytic zone around the colonies.

To test gelatin hydrolysis activity, a nutrient gelatin medium was employed. BST.L-601 broth and protease (positive control) (P5147, Sigma-aldrich, St. Louis, MO, USA) were inoculated with a concentration of 10 mg/mL and incubated for 7 days at 25 °C. The agar tubes were placed at 4 °C for 10 min to observe the change in its formulation by gelatinase.

To test biogenic amine (BA) productivity, Bover-Cid and Holzapfel medium agar containing amines (L-tyrosine, L-ornithine, and L-histidine with a concentration of 10 g/L, adjusted to pH 5.3) was employed. BST.L-601 and *L. rhamnosus* GG, as positive control strains, were streaked onto an agar plate and incubated for 48 h at 37 °C under anaerobic conditions. The BA productivity was assessed with changed colors, from yellow to purple by increased pH with BA, around the colonies.

### 2.3. Probiotic Properties Assessment

The characteristics, as lactic acid bacteria, of the strain BST.L-601 were verified. Bile salt tolerance assay, mucin degradation assay, and Caco-2 human colorectal cell adhesion assay were performed [27,28,29].

To test the bile salt tolerance of the strain, BST.L-601 and *L. rhamnosus* GG were streaked onto MRS agar containing 0.5% (*w*/*v*) taurodeoxycholic acid and then incubated for 48 h at 37 °C under anaerobic conditions.

To test mucin degradation activity, broth of BST-L.601, *L. rhamnosus* GG, and *Bacillus* spp. was dotted onto an agar plate containing 0.3% (*w*/*v*) mucin and then incubated for 48 h at 37 °C under anaerobic conditions. The activity was estimated with amido-black staining and acetate washing, followed by observation of mucin lysis around bacterial colonies.

To test the cell adhesion activity of BST.L-601, Caco-2 cells and *L. rhamnosus* GG were employed. The Caco-2 human colorectal cells (American Type Culture Collection; ATCC, Manassas, VA, USA) were cultured in high-glucose DMEM (Dulbecco’s Modified Eagle’s Medium; Hyclone, Logan Road, UT, USA) supplemented with 10% FBS (Fetal bovine serum; Corning, NY, USA) and 1% antibiotic solution (penicillin–streptomycin; Gibco, Grand island, MA, USA) at 37 °C in 5% CO_2_. The cells were seeded at a density of 1.0 × 10^5^ cells/well in a 12-well cell culture plate. After 12 h of culture, cell media were changed to the medium without antibiotics to prevent suppression of the bacteria and incubated for 2 h. Fermented BST.L-601 was centrifuged and washed with phosphate-buffered saline (PBS) and then diluted 10^4^ to 10^6^ times. Then, 0.1 mL of diluted BST.L-601 broth was mixed with 0.9 mL of the cell culture media and then inoculated to the caco-2 cell culture plate for 2 h. Non-adherent bacterial cells were eliminated with PBS, and adherent cells were collected using Triton X-100. The formed colonies of inoculated BST.L-601 and collected adherent cells after incubation with Caco-2 cells were compared using MRS agar plate spreading.

### 2.4. Fermentation

Strain BST.L-601 was subjected to a three-phase culture process, including an activation phase to activate the inoculated strain, a seed phase to increase the population, and a main culture phase with a 2 L jar fermenter. The main culture was carried in a broth as described in Table 1, with 0 or 2% sweet potato (SP) powder (Mungyeong-si, Republic of Korea), at 37 °C for 12 h with agitation. Additionally, the pH of the culture was adjusted to pH 5.8 using an ammonia solution during fermentation.

### 2.5. Stability Assessment

BST.L-601 cells were harvested through centrifugation (8000 rpm, 30 min, 25 °C) after the main culture and mixed with a cryoprotectant (Table S1) in equivalent weight of the cells from the main culture with 0% sweet potato powder. The mixture was freeze-dried, aliquoted into aluminum pouches, and stored in 3 different conditions; low (4 °C), room (25 °C), and extreme (35 °C) temperature.

The stored samples under each condition were freshly opened at 2-week intervals, and the number of CFU/g was determined through serial dilution. Serial dilution was performed with PBS and MRS agar (BD Difco, Franklin Lakes, NJ, USA), and 200 μL of each suspension was plated onto MRS agar. Plates were incubated at 37 °C for 48 h under anaerobic conditions.

### 2.6. Cell Viability

Cell viability was estimated using EZ-Cytox (DoGenBio, Seoul, Republic of Korea) to determine the effects of BST.L-601 on C2C12 mouse myoblast cells. The C2C12 mouse myoblast cells (ATCC, Manassas, VA, USA) were cultured in high-glucose DMEM supplemented with 10% FBS and 1% antibiotic solution at 37 °C in 5% CO_2_. After seeding myoblast cells at a density of 1 × 10^4^ cells/well in a 96-well cell culture plate, differentiation was induced 24 h later by changing the medium to DMEM containing 2% (*v*/*v*) horse serum for 5 days. The differentiated myotube cells were treated with heat-killed BST.L-601, diluted to desired concentrations (2.5 × 10^7^ to 1 × 10^9^ CFU/mL) using DMEM, for 24 h. Heat-killed BST.L-601 was prepared by heating PBS-suspended BST.L-601 at 121 °C for 20 min, followed by using its supernatant. After, the WST reagent was diluted 1/10 in cell culture medium, treated in each well, and reacted for 20 min in a 5% CO_2_ incubator at 37 °C. Cell viability was measured at an absorbance of 450 nm using a microplate reader (Spark multimode microplate reader, TECAN, Männedorf, Switzerland).

### 2.7. Glucose Uptake Assay

The glucose uptake in the C2C12 cells was measured using a fluorescent derivative of glucose called 2-NBDG (2-deoxy-2-[(7-nitro-2,1,3-benzoxadiazol-4-yl) amino]-D-glucose; Invitrogen, Waltham, MA, USA). The C2C12 cells were seeded in 24-well plates at a density of 5 × 10^4^ cells/well, and differentiation was induced after 24 h with DMEM medium containing 2% horse serum. The cells were treated with heat-killed BST.L-601 (5 × 10^7^ to 5 × 10^8^ CFU/mL) in glucose-free DMEM medium (Gibco, Grand Island, MA, USA) for 16 h. As a positive control group, insulin (100 nM) was treated under the same conditions. After, cells were incubated with 2-NBDG (50 µM) for 2 h and then washed with cold PBS. The cells were lysed with RIPA buffer, and the supernatant was transferred to a 96-well black plate. The fluorescence intensity of cellular 2-NBDG was measured at an excitation wavelength of 485 nm and an emission wavelength of 535 nm using a microplate reader.

### 2.8. α-Glucosidase Inhibition Assay

The α-glucosidase activity was measured using the method of Watanabe, which was slightly modified [30]. The α-glucosidase (0.4 unit/mL) and p-nitrophenyl-α-D-glucopyranoside (5 mM) were dissolved in 0.1 M phosphate-buffered solution (pH 7.0) containing 0.2% BSA (bovine serum albumin) and 0.02% sodium azide, respectively, to prepare enzyme and substrate solutions. The acarbose was used as a positive control [31]. The enzyme solution and BST.L-601 (1 × 10^7^ to 5 × 10^9^ CFU/mL) and acarbose (1 to 5 mg/mL) samples were incubated together in a 96-well plate at 37 °C for 10 min. Subsequently, PNPG (p-nitrophenyl-α-D-glucopyranoside, 5 mM) was added to the reaction mixture and incubated at 37 °C for 10 min. The reaction was terminated by adding 1 M sodium carbonate solution, and the enzymatic reaction was measured at an absorbance of 405 nm using a microplate reader. The α-glucosidase inhibition rate was calculated using the following equation:

$$\left(\alpha \text{-}\mathrm{glucosidase}\;\mathrm{inhibition}\;\mathrm{activity},\%\right)=\frac{Abs\;\left(\mathrm{blank}\;\mathrm{test}\right)-Abs\;\left(sample\right)}{Abs\;\left(\mathrm{blank}\;\mathrm{test}\right)}\;\times 100$$

### 2.9. Animal Care and Treatment

All animal procedures were approved by the Institutional Animal Care and Use Committee of the Catholic University of Korea (Approval No.: CUK-IACUC-2024-021, Date of Approval: 31 July 2024). All efforts were made to minimize animal use and distress. Male C57BL/KSJ and C57BL/KSJ db/db (Lepr^db^/Lepr^db^) mice (5 weeks old) were purchased from Central Lab Animal Inc. (Seoul, Republic of Korea) and housed at 22 °C under a 12 h light/dark cycle. Mice were acclimated for one week before the experiment and fed a restricted diet (5 g/mouse/day) of AIN-76A rodent diet (Research Diets, New Brunswick, NJ, USA).

C57BL/KSJ mice were assigned to the normal control group (C), and C57BL/KSJ db/db mice were divided into four groups (*n* = 5): NC (untreated control), BST.L-601-L (1.00 × 10^9^ CFU/kg/day), BST.L-601-H (2.00 × 10^9^ CFU/kg/day), and PC (positive control, metformin 250 mg/kg/day). All samples were dissolved in PBS and administered orally daily for 8 weeks. During the treatment period, body weight and water intake of each group were recorded weekly. The C and NC groups received the same volume of PBS. After the final administration, mice were fasted for 16 h (with free access to water) and then anesthetized with an intraperitoneal injection of the alfaxan–rompun mixture. Blood was collected through cardiac puncture, placed on ice for 30 min, and centrifuged at 13,000 rpm for 15 min at 4 °C. The supernatant (serum) was stored at −80 °C until use. Organs (liver, kidney, pancreas, and spleen) were surgically removed, weighed, frozen in liquid nitrogen, and stored at −80 °C. Skeletal muscle and feces were collected and stored at −80 °C. All physiological, biochemical, and histological parameters (including body weight, fasting blood glucose, oral glucose tolerance test (OGTT), serum analyses, and IHC) were measured in five mice per group (*n* = 5), whereas only the RT-PCR assays were conducted using three randomly selected representative samples per group (*n* = 3). The sample size (*n* = 5 per group) was determined based on previous studies employing db/db mouse models for evaluating anti-diabetic effects, which demonstrated adequate statistical power to detect significant differences in fasting blood glucose and OGTT outcomes [32].

#### 2.9.1. Blood Glucose Measurement

Fasting blood glucose (FBG) was measured once a week. Mice were fasted for 16 h before measurement, with free access to water. Blood glucose was determined from the tail vein using a Accu-CHEK blood glucose meter (Roche Diabetes Care GmbH, Mannheim, Germany).

An oral glucose tolerance test (OGTT) was performed in the final week of the experiment (week 8). Mice were fasted for 16 h and administered an oral glucose tolerance test solution (2 g glucose/kg). Blood glucose levels were measured at 0, 30, 60, 90, 120, 150, and 180 min using an Accu-CHEK blood glucose meter (Roche Diabetes Care GmbH, Germany).

#### 2.9.2. Serum Analysis

Collected blood serum samples were analyzed at T&P bio (Gwangju-si, Republic of Korea). Levels of alanine aminotransferase (ALP), aspartate aminotransferase (GOT), alkaline phosphatase (GPT), blood urea nitrogen (BUN), creatinine (CREA), blood glucose, triglyceride (TG), total cholesterol, high-density lipoprotein cholesterol (HDL), and low-density lipoprotein cholesterol (LDL) were measured using an automatic analyzer (AU680; Beckman Coulter, Brea, CA, USA).

#### 2.9.3. Reverse Transcription–Polymerase Chain Reaction (RT-PCR)

Total RNA was extracted using an Easy-spinTM Total RNA Extraction Kit (iNtRON Biotechnology, Seongnam-si, Republic of Korea), according to the manufacturer’s instructions. After synthesis of complementary DNA (cDNA) using a High Capacity cDNA Reverse Transcription Kit (Applied Biosystems, Waltham, MA, USA), PCR was performed using a Maxime PCR PreMix Kit (iNtRON Biotechnology, Seongnam-si, Republic of Korea) with proper sense and antisense primers for the IRS-1 gene (IRS-1; sense primer, 5′-TGC TCA ATA GCG TAA CTG GA-3′; antisense primer, 5′-AGA ACG TGC AGT TCA GTC AA-3′), GLUT4 gene (GLUT4; sense primer, 5′-ACA TAC CTG ACA GGG CAA GG-3′; antisense primer, 5′-CGC CCT TAG TTG GTC AGA AG-3′), and β-actin gene (β-actin; sense primer, 5′-TGC TGT CCC TGT ATG CCT CT-3′; antisense primer, 5′-AGG TCT TA CGG ATG TCA ACG-3′) under the following conditions; IRS-1, 33 cycles of denaturation at 95 °C for 30 s, annealing at 54.6 °C for 30 s, and extension at 72 °C for 30 s; GLUT4, 30 cycles of denaturation at 95 °C for 60 s, annealing at 59 °C for 30 s, and extension at 72 °C for 30 s; β-actin, 33 cycles of denaturation at 94 °C for 20 s, annealing at 60.6 °C for 10 s, and extension at 72 °C for 30 s. PCR products were resolved on 1.5% agarose gels, stained with EcoDye nucleic acid staining solution (ES-301; Biofact, Daejeon-si, Republic of Korea), and visualized under UV transillumination. RT-PCR band intensity was quantified using ImageJ (Version 1.54f), and brightness/contrast were uniformly adjusted across all samples for visualization.

#### 2.9.4. Histological Analysis

Liver and pancreas tissues were collected, and histological analysis was conducted by T&P bio (Gwangju-si, Republic of Korea). Collected tissues were fixed in 10% (*v*/*v*) formalin solution and embedded in paraffin. Hematoxylin and eosin (H&E) staining was performed to compare the morphological change in the liver and the pancreas. Immunohistochemistry (IHC) staining was performed with the primary antibody (Cell Signaling Technology, Inc., Danvers, MA, USA) against insulin (4590S, 1:400) and glucagon (2760S, 1:100) to confirm the effect of BST.L-601 on glucagon production of alpha cells and insulin production of beta cells in the pancreas islets. For imaging of stained slides, a slide scanner (APERIO CS2, Leica, Germany) and viewing software (Aperio ImageScope 12.4.6, Leica, Germany) were used.

#### 2.9.5. Gut Microbiota Analysis

The alpha and beta diversity analyses were conducted according to the guidelines of Theragen Bio (Seongnam-si, Republic of Korea) to determine the diversity of intestinal microorganisms. Stool samples from the large intestine of mice were used for diversity analyses (*n* = 3). First, 100 ng of genomic DNA for a 350 bp insert size was fragmented using a Covaris S2 Ultrasonicator (Covaris, Woburn, MA, USA). DNA sequencing libraries were constructed using the Truseq Nano DNA library prep kit (Illumina, San Diego, CA, USA), according to the manufacturer’s protocol. The quality of amplified libraries was confirmed through electrophoresis using an Agilent Bioanalyzer High Sensitivity DNA Kit (Agilent, Santa Clara, CA, USA). Libraries were quantified using the KAPA Library Quantification Kit (Kapa Biosystems, Wilmington, MA, USA), according to the manufacturer’s library quantification protocol. Following cluster amplification of denatured templates, sequencing was progressed as paired-end (2× 150 bp) using Illumina Novaseq X plus (Illumina, San Diego, CA, USA).

#### 2.9.6. Statistical Analysis

Statistical analyses were performed using GraphPad Prism5 (Graph Pad Software Inc., San Diego, CA, USA). Statistical significance for all parameters was determined using a one-way analysis of variance (ANOVA) test, followed by Dunnet’s multiple comparisons test. Unless otherwise stated, all data from triplicate measurements are presented as the mean ± SD. A *p*-value of <0.05 was considered statistically significant.

## 3. Results

### 3.1. Safety of the Strain BST.L-601

To start with, the strain of BST.L-601 was clearly confirmed as a representative probiotic strain *Lacticaseibacillus rhamnosus,* through 16S rRNA sequencing (Figures S1 and S2). As probiotics are functional health foods intended for human consumption, ensuring their safety must be given the highest priority [33]. Accordingly, antibiotic susceptibility, existence of virulence genes, hemolytic activity, gelatin hydrolysis activity, and potential of biogenic amine production of BST.L-601 were investigated (Table 2).

First, as shown in Table 2, all minimal inhibitory concentration (MIC) values for BST.L-601, including the representative strain *L. rhamnosus* GG, measured below the European Food Safety Authority (EFSA) standard cut-off value (Table S2). Additionally, screening of virulence genes with the VirulenceFinder 2.0. program showed an absence of virulence-associated genes in the assembled genomic data. This suggests that the sequenced sample did not contain known virulence factors detectable using the VirulenceFinder 2.0. database and algorithm (Table S3).

For the assessment of hemolytic activity, BST.L-601 and *L. rhamnosus* showed no change around the colonies in the blood agar plate (γ-hemolysis) (Figure S3), while the positive control strain *E. coli* showed a clear zone around the colonies through lysis of red blood cells (β-hemolysis) (Figure S3C), indicating the non-hemolytic activity of BST.L-601.

To assess gelatin hydrolysis, gelatin was lysed to liquid with the addition of protease (Figure S4A), while gelatin-inoculated BST.L-601 (Figure S4B) and the inoculated medium (Figure S4C) maintained their solid states.

To determine the productivity of biogenic amine of BST.L-601, BST.L-601 and *L. rhamnosus* GG showed no change to purple, implying increased pH with produced biogenic amine by the strain (Figure S5).

With these results, it is confirmed that the strain *L. rhamnosus* BST.L-601 satisfies the EFSA standards for antibiotic susceptibility, with an absence of hemolytic activity and gelatin hydrolytic activity and no potential for biogenic amine production.

### 3.2. Probiotic Properties Assessment

All strains grew and created colonies with no change (Figure S6A,C) compared to the colonies on MRS agar without taurodeoxycholic acid (Figure S6B,D), showing that BST.L-601 has bile tolerance.

To estimate mucin lysis activity, two control strains, *L. rhamnosus* and *Bacillus* spp., were employed as the positive and negative controls. Mucin lysis around *Bacillus* spp. colonies was observed, with a thin layer of clear zone (Figure S4C), while no lysis was observed for the colonies of BST.L-601 and *L. rhamnosus* GG (Figures S4A–S7B).

Moreover, BST.L-601 showed remarkable adhesion ability (Figure S8A,B), compared to *L. rhamnosus* GG (Figure S8C,D).

These experiments demonstrate the probiotic properties of BST.L-601, including bile salt tolerance, colorectal cell adhesion activity, and the absence of mucin lysis activity. This confirms that the strain is suitable for industrial use.

### 3.3. The Effect of Sweet Potato Powder on the Culture of BST.L-601

**Figure 1:**
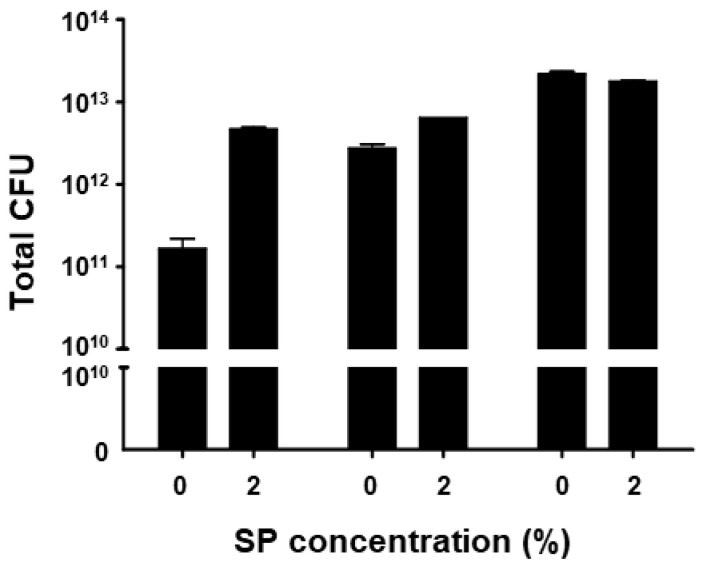
Measured CFU of 3 batches of BST.L-601 culture with 0 and 2% of sweet potato (SP) concentrations. Values are the means ± SD.

Fermentation of BST.L-601 showed greater viability with a culture including 2% sweet potato (SP) powder (Figure 1). Cultures were carried out in triplicate for each medium condition, and the mean values of the resulting live bacterial counts of freeze-dried broth (CFU/g) were compared. As shown in Figure 1, a higher CFU was shown in the culture with media including 2% sweet potato powder compared to media without sweet potato powder. BST.L-601 had the highest viable count at 12 h of culture.

### 3.4. Stability Assessment

**Figure 2:**
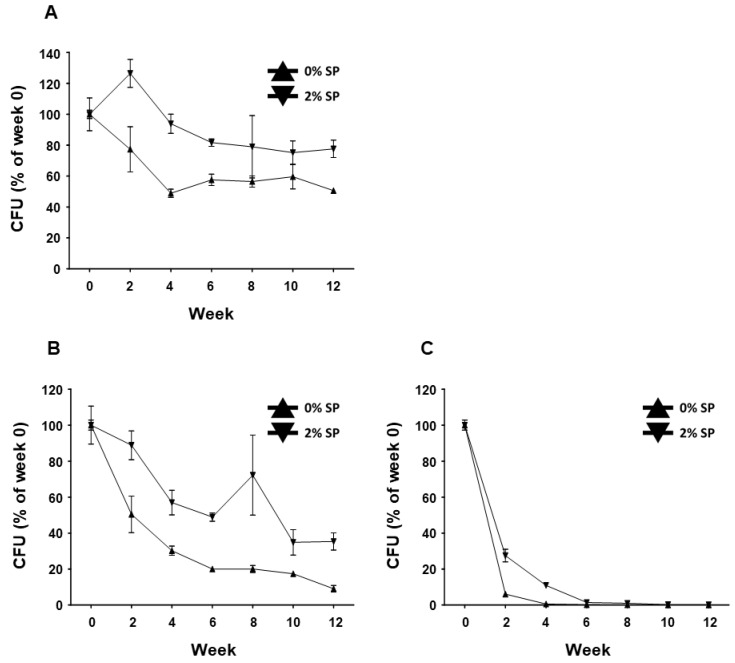
Stability assessment of BST.L-601 under 3 temperature conditions. Measured CFU/g, expressed as % of week 0, of BST.L-601 stored at (**A**) 4 °C, (**B**) 25 °C, and (**C**) 35 °C. Values are the means ± SD.

BST.L-601 fermented with 0 and 2% SP powder was collected from the broth, mixed with cryoprotectant, freeze-dried, and pulverized into powder. The powdered BST.L-601 was aliquoted into 1 g portions and stored in silver foil pouches in 3 different conditions (4, 25, and 35 °C). The CFU of stored samples in each condition was measured every 2 weeks. Each sample was subjected to serial dilution with phosphate-buffered saline with Tween 80 (PBST, pH 7.0, 0.1% (*w/v*) Tween 80), spread onto an MRS agar plate, and incubated for 48 h under anaerobic conditions. The CFU of live bacteria from each sample was measured, and the stability of BST.L-601 was assessed (Figure 2). BST.L-601 fermented with 2% SP powder showed higher CFU than BST.L-601 fermented with 0% SP powder in all three temperature conditions. Samples of BST.L-601 with 2% SP maintained a stable CFU (>50%) for 12 weeks at a low temperature (4 °C) (Figure 2A) and a CFU > 60% for 4 weeks at room temperature (25 °C) (Figure 2B). In extreme conditions (35 °C), BST.L-601 with 2% SP showed a CFU decreased to less than 20% from the second week of storage (Figure 2C).

**Figure 3:**
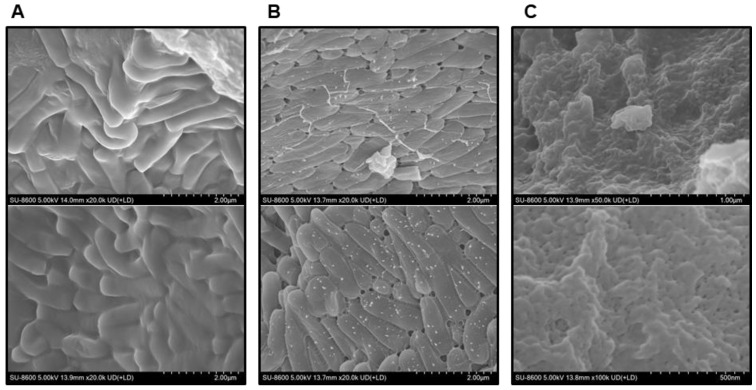
Observation of powdered sample of BST.L-601 using SEM (HITACHI SU8600). (**A**) BST.L-601 fermented with media with industrial composition, 20,000×. (**B**) BST.L-601 fermented with industrial media added 2% SP powder, 20,000×. (**C**) Industrial media added 2% SP powder, 50,000×.

**Figure 4:**
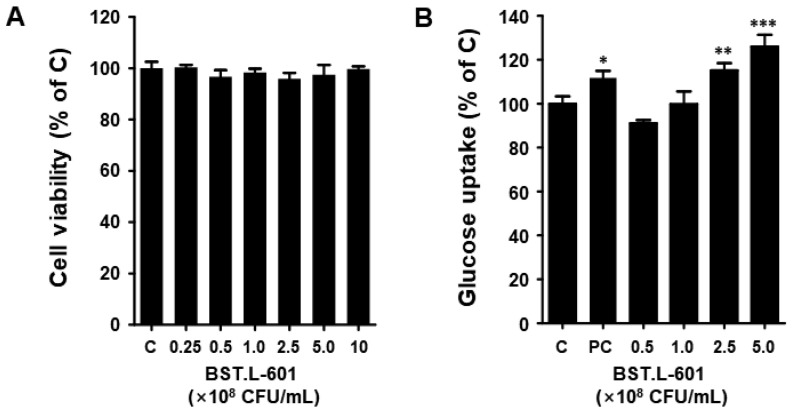
Cell viability, glucose uptake of BST.L-601 in C2C12 myotube. (**A**) Cell viability, (**B**) Glucose uptake. The data are expressed as the percentage normalized to the untreated control cells (100%). C, untreated; PC, positive control (insulin); Values are the means ± SD (n = 3), determined by one-way ANOVA and Dunnett’s multiple comparison test (* *p* < 0.05, ** *p* < 0.01, *** *p* < 0.001).

Subsequently, scanning electron microscopy (SEM) (SU8600, HITACHI, Tokyo, Japan) was conducted to infer the effect of the presence or absence of SP powder on the stability of BST.L-601 (Figure 3). Fermented BST.L-601 with identical media composition containing 0 or 2% SP powder and media with 2% SP powder were observed. As shown in Figure 4B, small particle-like substances on the surface of the cells were observed (Figure 3B), in contrast to those without SP powder (Figure 3A).

### 3.5. Cell Viability Assay of C2C12

Prior to assessing the anti-diabetic activity of BST.L-601 in vitro, the cell cytotoxicity of the heat-killed BST.L-601 was analyzed.

The C2C12 myoblast cells were cultured in a 96-well plate, and differentiation was induced. Cell viability was evaluated through treatment with 2.5 × 10^7^ to 1 × 10^9^ CFU/mL concentrations of heat-killed BST.L-601 for 24 h. In C2C12 myotube cells, the BST.L-601 sample showed more than 95% cell viability at all concentrations (Figure 4A). This suggests that BST.L-601 does not exhibit toxicity in C2C12 myotube cells up to a concentration of 1 × 10^9^ CFU/mL.

### 3.6. Glucose Uptake Assay of C2C12

The 2-NBDG assay was performed to analyze the effect of BST.L-601 on intracellular glucose inflow. This experiment evaluated the effect of BST.L-601 on glucose uptake in differentiated C2C12 cells. When differentiated C2C12 cells were treated with insulin, glucose uptake increased, and when treated with BST.L-601, glucose uptake also increased in a dose-dependent manner (Figure 4B). In particular, the group at a concentration of 2.5 × 10^8^ CFU/mL of BST.L-601 showed increased glucose uptake (115%) similar to the group treated with insulin (111%), an anti-diabetic drug; at the highest treatment concentration (5 × 10^8^ CFU/mL), it showed a higher glucose uptake rate (125%) than insulin (Figure 4B). Therefore, BST.L-601 may help with diabetes by reducing blood glucose levels by increasing glucose uptake in myotube cells.

### 3.7. α-Glucosidase Inhibitory Activity

**Figure 5:**
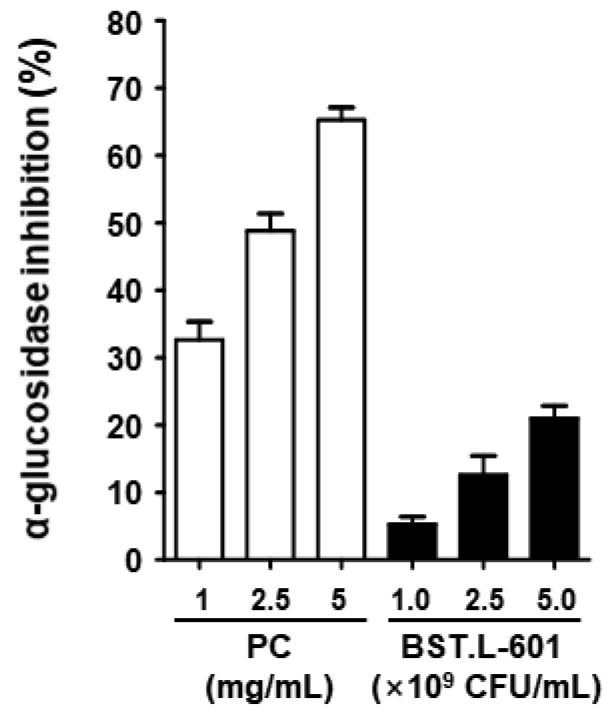
α-Glucosidase inhibitory activities of BST.L-601. The acarbose was used as a positive control (PC). Values are the means ± SD (*n* = 3).

The α-glucosidase inhibitory activity of BST.L-601 was evaluated. The inhibitory activity of BST.L-601 and the positive control, acarbose, was shown to be dose-dependent. Approximately 21% α-glucosidase inhibitory activity was shown at the highest treatment concentration of BST.L-601 (Figure 5). Although the inhibitory activity of BST.L-601 was lower than that of acarbose, which is used as a diabetes treatment, it appears to have the potential to be utilized as a material for improving diabetes.

### 3.8. Effects of BST.L-601 on the T2DM-Induced Mouse Model

#### 3.8.1. Care and Treatment of Mice Fed Different Diets

**Figure 6:**
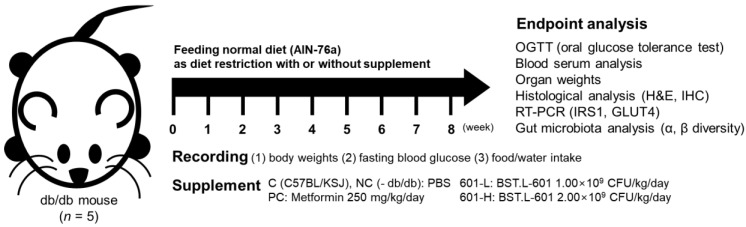
Scheme for animal treatment and analyses conducted after 8 weeks of administration.

Male C57BL/KSJ mice were prepared as a control group, C, and C57BL/KSJ db/db (Lepr^db^/Lepr^db^) mice were divided into 4 experimental groups (*n* = 5): NC, untreated control; PC, positive control (metformin 250 mg/kg/day); 601-L, freeze-dried BST.L-601 broth at low concentrations (1.00 × 10^9^ CFU/kg/day); 601-H, freeze-dried BST.L-601 broth at high concentrations (2.00 × 10^9^ CFU/kg/day) (Figure 6). Oral administration was prepared with each sample dissolved in PBS and performed daily. Body weight and water intake changes were recorded once a week, with free access to water. Fasting blood glucose (FBG) changes were recorded biweekly, after a 16 h of fast.

#### 3.8.2. Body Weight and Blood Glucose Changes in Mice Fed BST.L-601

After 8 weeks of oral administration, none of the groups showed a significant difference in body weight (Figure S9A), including the untreated group (28.79 g), the positive control group (31.13 g), and the BST.L-601-treated group (601-L, 26.19 g; 601-H, 30.92 g). There were no significant differences in the body weights of the 4 groups; however, the PC group, which included metformin-treated mice, and the 601-H group, which comprised mice treated with high doses of BST.L-601, demonstrated the highest values, which were similar.

For water intake, intake volume showed a decreasing trend in the order of PC, NC, 601-H, and 601-L, while the measured volumes were comparable at the beginning (weeks 1–2) and end of treatment (weeks 7–8) (Figure S9B).

As shown in Figure S9C, the untreated group (NC) showed the highest fasting glucose level (449 mg/dL), and the positive control group showed a decreased level (423.4 mg/dL). The BST.L-601-treated group showed a dose-dependent decrease in the fasting glucose level by 405.6 mg/dL (601-L) and 395.4 mg/dL (601-H). Along with biweekly measurements of fasting blood glucose, an oral glucose tolerance test (OGTT) was carried out during the final week (Figure S9D). At the final 180 min measurement, the untreated group (NC) showed similar blood glucose values (593.6 mg/dL) to the 601-L group (592.6 mg/dL). The PC group (543.6 mg/dL) and the 601-H group (521.4 mg/dL) showed decreased values compared to the NC group. These results suggest that BST.L-601 has blood-lowering activity in a T2DM model mouse model in a dose-dependent manner.

Furthermore, organ weight changes in groups were compared after sacrifice (Table 3). Kidneys, the pancreas, and the liver showed comparable differences in weight, but the spleen showed decreased weight in all groups compared to the untreated group (NC). The NC group had the highest spleen weight, and the BST.L-601-treated group with a low concentration (601-L) had the lowest spleen weight.

#### 3.8.3. Serum Analysis

To assess the effects of BST.L-601 on mice, serum component levels of alanine aminotransferase (ALP), aspartate aminotransferase (GOT), alkaline phosphatase (GPT), blood urea nitrogen (BUN), creatinine (CREA), blood glucose, triglyceride (TG), total cholesterol, high-density lipoprotein cholesterol (HDL), and low-density lipoprotein cholesterol (LDL) were analyzed.

Levels of indicators of liver damage or disease, ALP, GOT, and GPT, showed no marked change between the groups (Table 4). However, for the BST.L-601 treatment, the values for each factor increased from the low-concentration to the high-concentration treatment groups.

Levels of BUN and creatinine, indices that reflect renal function, showed no notable difference between the untreated group (NC) and the BST.L-601-treated groups (601-L, 601-H), except for the metformin-treated group (PC), and the PC group showed higher BUN values compared to the NC group (Table 4).

Levels of serum glucose and triglyceride, typical body metabolism indices, decreased with BST.L-601 treatment in a dose-dependent way (Table 4). Treatment with metformin (PC) did not have any impact.

Levels of total cholesterol, HDL, and LDL were analyzed, as well (Table 4). Compared to the untreated group (NC), the positive control (PC) and BST.L-601-administered groups (601-L, 601-H) showed increased HDL levels, while the LDL level of each group showed no significant change. Consequently, the ratio value of LDL/HDL improved.

#### 3.8.4. Reverse Transcription–Polymerase Chain Reaction (RT-PCR)

To assess the anti-diabetic effects of BST.L-601 on the mRNA expression levels of insulin-signaling-related biomarkers in T2DM-induced mice, RT-PCR analysis was conducted. After 8 weeks of BST.L-601 administration, skeletal muscle tissues were lysed, and the amount of mRNA was measured. The mRNA expression levels of IRS-1 (Insulin receptor substrate 1) and GLUT4 (Glucose transporter 4) were measured.

**Figure 7:**
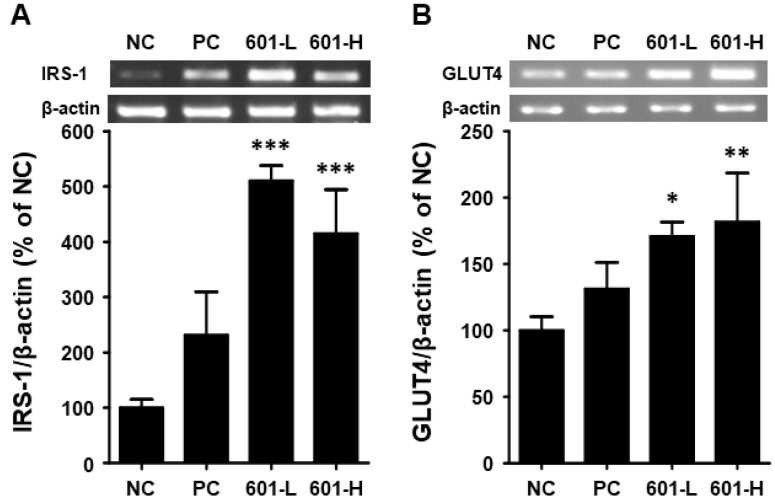
Effects of BST-L.601 on the mRNA expression of IRS-1 and GLUT4 in skeletal muscle of db/db mice. Skeletal muscle tissues from db/db mice treated with BST-L.601 for 8 weeks were homogenized and processed for RT-PCR analysis. The relative mRNA levels of (**A**) IRS-1 and (**B**) GLUT4 were quantified using ImageJ software (Version 1.54f). NC, untreated group; PC, metformin-treated group; 601-L, low concentration of BST.L-601-treated group; 601-H, high concentration of BST.L-601-treated group. Values are the means ± SD (*n* = 3), determined by one-way ANOVA and Dunnett’s multiple comparison test (* *p* < 0.05, ** *p* < 0.01, *** *p* < 0.001).

As shown in Figure 7, the administration of BST.L-601 increased the expression level of IRS-1 by 510.20% (601-L) and 414.65% (601-H) and that of GLUT4 by 170.86% (601-L) and 181.86% (601-H).

#### 3.8.5. Histological Analysis

**Figure 8:**
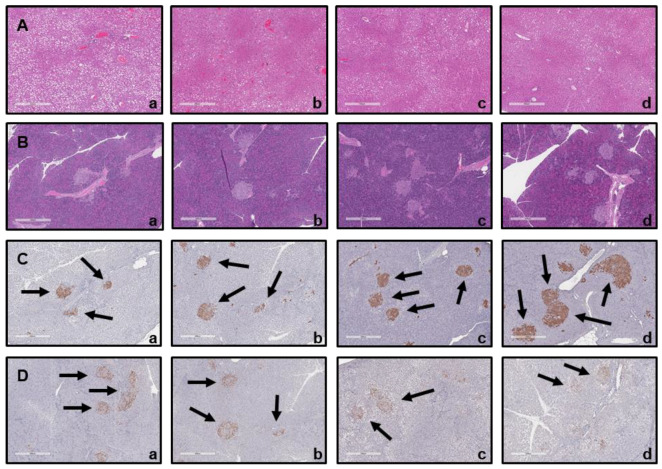
Histological analysis of mice following oral administration of BST.L-601. Hematoxylin and eosin (H&E) staining of (**A**) liver and (**B**) pancreas; (**a**) NC, (**b**) PC, (**c**) 601-L, (**d**) 601-H. Immunohistochemical (IHC) staining of (**C**) β-cell with insulin antibody (Black arrows) and (**D**) α-cell with glucagon antibody (Black arrows); (**a**) NC, (**b**) PC, (**c**) 601-L, (**d**) 601-H. NC, untreated group; PC, metformin-treated group; 601-L, low concentration of BST.L-601-treated group; 601-H, high concentration of BST.L-601-treated group.

The size of the liver (Figure 8A) and pancreatic islet cells (Figure 8B) was comparable across all groups, including the untreated group (NC), the positive group (PC), and the BST.L-601-treated group (601-L, 601-H). Furthermore, compared to the NC group, the size of insulin-producing beta cells was increased in the PC and BST.L-601-treated groups (601-L, 601-H) (Figure 8C). Moreover, compared to the NC group (b), the 601-L and 601-H groups (c, d) exhibited a significant decrease in the size of alpha cells producing glucagon (Figure 8D).

#### 3.8.6. Effect of BST.L-601 Treatment on Microbial Diversity

**Figure 9:**
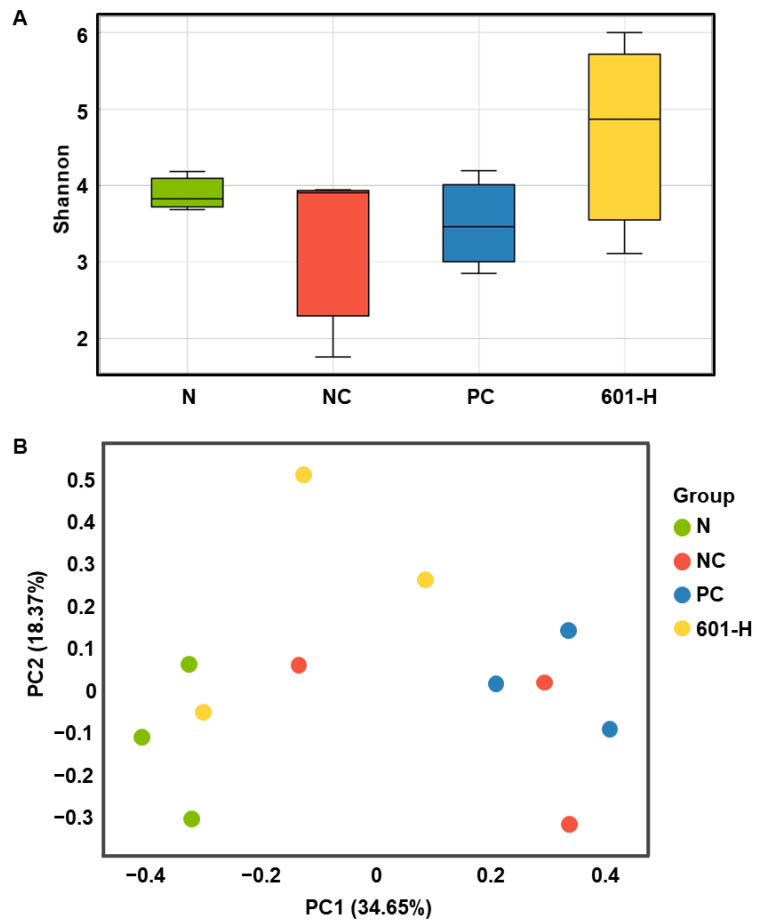
Analysis of gut microbiome diversity. (**A**) Box plot for Shannon alpha diversity and (**B**) Principal Coordinates Analysis. Values are the means ± SD (*n* = 3), determined by one-way ANOVA and Dunnett’s multiple comparison test.

Alpha diversity and beta diversity are used to assess microbial diversity. Alpha diversity is an analytical method for assessing the diversity within a sample. A higher value indicates a greater diversity of microorganisms within the sample. The differences in microbial diversity between groups were confirmed using a Shannon alpha diversity box plot (Figure 9A). The BST.L-601 group had higher values than the N (normal), NC (negative control), and PC (positive control) groups, confirming a more diverse microbial community (Figure 9A). Beta diversity is an analytical method used to determine the diversity between samples, and it is used when comparing the diversity of microbial communities in two or more samples. The Bray–Curtis dissimilarity analysis takes a value between 0 and 1, depending on species abundance. A value closer to 0 indicates the same species among samples, while a value closer to 1 indicates no common species among samples. The beta diversity results were expressed using PCoA (Principal Coordinates Analysis). The PCoA is an analytical method that visualizes multivariate data in a low-dimensional space to intuitively understand the similarity between samples. This means that samples that are close to each other have high similarity, and samples that are far apart have low similarity. The NC group and the PC group have similar cluster structures because the distance between the two sample points is close, while the N group and the NC and PC groups have different cluster structures and can be interpreted because the distance between the two sample points is far. And although the BST.L-601 group has samples that are close to the N, NC, and PC groups, when evaluated as an overall sample cluster, the distance between clusters is large, which can be interpreted as a difference in diversity between groups (Figure 9B).

## 4. Discussion

Type 2 diabetes mellitus (T2DM) is a disease caused by a combination of insulin resistance, the body’s inability to properly utilize insulin, and a decline in the body’s ability to secrete insulin. Furthermore, increased cellular insulin resistance leads to hyperglycemia and decreased cellular sensitivity to insulin. The present study assessed the anti-diabetic effects of the probiotic strain *Lacticaseibacillus rhamnosus* BST.L-601, especially in T2DM, and evaluated the improved productivity and storage stability of fermented BST.L-601 with sweet potato (SP) as a medium component. Through in vitro and in vivo assays, the use of BST.L-601 as an anti-diabetic synbiotic material in the functional food industry was supported.

First, the species of BST.L-601 was verified through 16S rRNA sequencing and identified as *Lacticaseibacillus rhamnosus* (Figures S1 and S2), with its various advantages, and a lot of strains have been discovered from [34].

Because BST.L-601 is anticipated to be used as a functional probiotic strain for human consumption, its safety is paramount. Antibiotic susceptibility is important to ensure the safety of the strain and prevent the spread of antibiotic resistance genes (ARG), which could lead to health problems [35]. Therefore, it was necessary to confirm whether BST.L-601 is resistant to specific antibiotics through an antibiotic susceptibility test. As shown in Table S2, BST.L-601 had antibiotic susceptibility values below the European Food Safety Authority (EFSA) standard for *L. rhamnosus* using a minimum inhibitory concentration (MIC) test. Moreover, whole genome sequencing (WGS) for certifying the absence of virulence genes (Table S3) and assays for hemolytic activity, gelatin hydrolysis activity, and biogenic amine (BA) productivity demonstrated the safety of BST.L-601 (Table 2 and Figures S3–S5). These results showed that BST.L-601 is free from toxicological concerns and suitable for use in the food industry.

Therefore, this strain does not have hemolytic toxins, suggesting that it has low or no pathogenicity. Given its lack of gelatinase and biogenic amine, substances related to toxicity, its safety was confirmed.

Furthermore, supplementary assays were conducted to confirm the functionality of BST.L-601 as a probiotic strain to ascertain its potential for more effective utilization as a functional health food. BST.L-601 demonstrated bile tolerance (Figure S6), showing its ability to survive the human gastrointestinal tract when consumed. Furthermore, BST.L-601 did not degrade mucin (Figure S7), indicating that it will protect the mucosal barrier.

With the assurance of the safety and probiotic characteristics of BST.L-601, the effects of sweet potato powder (SP) in the fermentation of BST.L-601 were assessed. BST.L-601 was fermented with a medium supplemented with 0 and 2% SP (BST.L-601-0% SP, BST.L-601-2% SP). Through the fermentation process, productivity and storage stability according to the concentration of SP were evaluated. The total CFU of each fermentation and the average CFU of 3 batches, with 0 and 2% SP in the medium, were measured and compared (Figure 2). CFU comparison showed the enriched viability of BST.L-601-2% SP by 16.32% compared to the BST.L-601-0% SP. In the evaluation of stability in 3 different storage conditions (4, 25, and 35 °C), freeze-dried BST.L-601-0% SP and BST.L-601-2% SP were stored individually, and the CFU of each sample was measured in 2-week intervals. The 3 different temperatures were considered representative of the low, room, and high temperatures that could be encountered during distribution and storage in the industrialization of probiotics. 12 weeks of measurement showed remarkable results. BST.L-601-2% SP had a higher CFU than BST.L-601-0% SP across all evaluation periods in each of the three storage conditions (Figure 2). In particular, BST.L-601-2% SP maintained a CFU above 50% of the initial CFU in 4 °C (Figure 2A). In 25 °C, the CFU was >50% until week 8 (Figure 2B), while the CFU in 35 °C declined rapidly over 4 weeks (Figure 2C). These results indicate that SP has a significant role in BST.L-601 fermentation, improving bacterial viability and storage stability for industrial application. To assess the influence of SP on BST.L-601, scanning electron microscopy (SEM) was conducted (Figure 3). Small particle-like substances on the bacteria appeared under SEM observation of BST.L-601-2% SP (Figure 3B). Considering that sweet potato has plentiful nutrients, mainly starch and insoluble dietary fiber, the small particle-like substances were assumed to be starch or dietary fiber from SP [36]. A review of preceding studies indicates that the nutritional components of SP might provide protection against external environmental factors, thus promoting bacterial growth and enhancing storage stability [37,38]. Previous studies have shown the development of fermentation characteristics in probiotic strains using starch and fibers, improved *L. plantarum* biomass and storability using starch from plants (*Cassava* and *Bactris Gasepaes Kunth* palm), and enhanced *L. casei* viability during production, storage, and digestion using apple fibers [37,38].

The fermented product of BST.L-601 using SP was assessed to study its anti-obesity properties under the designation “SPY (Sweet Potato Yogurt)” [22]. Based on these findings, the authors decided to investigate its potential efficacy against diabetes, which is associated with obesity [39].

First, the anti-diabetic effect of BST.L-601 was examined using an in vitro assay with C2C12 myoblast cells. Treatment of BST.L-601 with a C2C12 myotube showed higher glucose uptake than insulin, a pharmaceutical anti-diabetic agent, with no cytotoxicity (Figure 4). Given that the myotube is a part of the muscle that absorbs blood glucose and uses it as an energy source, this suggests that BST.L-601 can reduce blood glucose [40]. Additionally, BST.L-601 showed α-glucosidase inhibition in a dose-dependent manner (Figure 5), demonstrating the potential to control blood sugar levels by slowing the breakdown and absorption of carbohydrates and preventing a rapid rise in blood glucose levels after food consumption. This inhibitory mechanism is consistent with the pharmacological action of acarbose and other α-glucosidase inhibitors used for type II diabetes management [41].

Subsequently, the anti-diabetic effect of BST.L-601 was inspected using an in vivo assay with a T2DM-induced db/db (Lepr^db^/Lepr^db^) mouse model. Over 8 weeks of oral administration of BST.L-601, the body weight of each group showed a tendency to decrease by 8 weeks, with comparable weight differences between the untreated group (NC) and the group treated with BST.L-601 (601-L) (Figure S9A). Water intake volume showed a decreasing trend during the intermediate phase (weeks 3-6) for BST.L-601-treated groups (601-L, 601-H) compared to the untreated group (NC) and the positive control group (PC, metformin-treated group) (Figure S9B). These differences suggest that induced T2DM in the NC and PC groups caused abnormal metabolism, resulting in increased water intake, while BST.L-601-administered groups (601-L, 601-H) showed decreased intake volume upon improving T2DM [42].

This inference was supported by biweekly measured fasting blood glucose (FBG) tests and oral glucose tolerance tests (OGTTs). Biweekly measurements of FBG revealed that the BST-L.601 treatment reduced blood glucose in a dose-dependent manner after 8 weeks (Figure S9C). Compared to the NC, both 601-L and 601-H lowered FBG levels, with the greatest reduction observed in the 601-H group. These results suggest that BST-L.601 exhibits blood-glucose-lowering effects similar to those of metformin, a well-established anti-diabetic agent.

Metformin is a biguanide agent that has been used as a first-line treatment for T2DM worldwide [43]. Among various diabetes medications, metformin was chosen as a positive control in the present study because of its treatment mechanism [44]. It improves T2DM by increasing insulin sensitivity and reducing glucose produced by the liver, rather than raising the amount of insulin in the body [44]. Given that T2DM is associated with an impaired ability to use insulin, metformin was determined to be appropriate for use in this study to compare the effects of curing induced T2DM in mice, rather than insulin secretagogues and other types of medication.

In the OGTT conducted at week 8, the BST.L-601-treated groups (601-L, 601-H) showed improved glucose tolerance compared to the NC group. In particular, the 601-H group demonstrated comparable glucose-lowering effects to metformin (Figure S9D). This suggests that BST-L.601 may enhance glucose utilization and insulin sensitivity in skeletal muscle, consistent with its effects on IRS-1 and GLUT4 expression described elsewhere in this study.

A similar result was demonstrated by the serum analysis (Table 4). Glucose and triglycerides (TG) levels were both decreased in a dose-dependent manner by the BST.L-601 intervention (Table 4), while high-density lipoprotein (HDL) levels increased (Table 4). These results indicate that BST.L-601 could improve the metabolism of glucose and lipids.

Enhanced metabolism was also demonstrated at the mRNA expression level. RT-PCR using mouse skeletal muscle displayed the intensified expression of glucose-metabolism-related factors, insulin receptor substrate-1 (IRS-1) and glucose transporter 4 (GLUT4) (Figure 7). In T2DM, insulin resistance prevents proper functioning [40]. This leads to dysfunction of the GLUT4 protein, which regulates blood sugar levels by transporting glucose into muscle and fat cells under the direction of insulin [45]. However, in patients with T2DM, insulin signals fail to properly induce GLUT4 transport across the cell membrane, preventing blood sugar from entering the cells [46]. This reduces glucose uptake by muscle and fat cells, leading to excessive accumulation of glucose in the blood, resulting in persistent hyperglycemia [46]. IRS-1 is a key protein in the insulin signaling pathway, regulating glucose uptake through activation of the insulin receptor [47]. When insulin binds to the insulin receptor, IRS-1 becomes phosphorylated and interacts with it. Phosphorylated IRS-1 activates the PI3K pathway, promoting the transportation and activation of the intracellular glucose transporter GLUT4, thereby facilitating glucose uptake into cells [45]. However, in T2DM patients, the phosphorylation (tyrosine phosphorylation) of IRS-1 is impaired, resulting in defective insulin signaling [48]. Abnormal phosphorylation of IRS-1 interferes with PI3K pathway activation, ultimately impairing glucose uptake and increasing blood sugar levels [40].

Based on a comparison of organ weight between mice groups and histological analyses of the liver and the pancreas, the cause of improved diabetes biomarkers was hypothesized (Table 3, Figure 8). After sacrificing the mice, organ weight measurements, hematoxylin and eosin (H&E) staining, and immunohistochemistry (IHC) were performed. The spleen and the pancreas exhibited distinct differences in weight between groups (Table 3). Pancreas weight was highest in the untreated group (NC), followed by mice treated with metformin (PC) and BST.L-601 (601-L, 601-H), in which the weight declined. In particular, pancreases of the 601-L group weighed less than those of the PC group, while spleen weights were similar. Consistent with the established correlation between murine inflammation and splenomegaly, the increased weight of the spleen and the pancreas of the NC group was proposed to result from the inflammatory response caused by induced diabetes in the mouse model [49,50]. It is also suggested that lower organ weight implies an improvement in diabetes with a subsequent anti-inflammatory effect through treatment with metformin (PC), a pharmaceutical agent for diabetes, or BST.L-601 interventions (601-L, 601-H). This improvement was further supported by the histological analysis of mice tissues (Figure 8). In a mouse model induced with type 2 diabetes, it was confirmed that β cells in the pancreatic islets of mice group fed BST.L-601 (601-L, 601-H) regenerated and recovered their function, resulting in increased insulin secretion, and glucagon secretion was reduced in α cells that increase blood sugar levels.

A decrease in microbial diversity can lead to an imbalance in the gut environment, increasing intestinal inflammation and insulin resistance and increasing the risk of developing diabetes [51]. Therefore, increasing gut microbiota diversity can have a positive impact on diabetes prevention and management [51,52]. Consuming specific microbes or fermented foods containing them, as well as fecal microbiota transplantation (FMT), can have anti-diabetic effects [53]. In the present study, the mouse gut microbial diversity of the BST.L-601-treated group was evaluated using alpha diversity analysis (Figure 9A), which evaluates diversity within a sample, and beta diversity analysis (Figure 9B), which measures diversity between samples. The α diversity analysis (Figure 9A) displayed that BST.L-601 administered mice group (601-H) has a wider range of intestinal microbial flora than the N (normal group), NC (diabetes induced group), and PC (metformin treated group). This result suggested that the consumption of BST.L-601 induced proliferation of probiotic microorganisms. Additionally, the β diversity analysis (Figure 9B) showed that the bacterial community diversity in the 601-H group differed from NC and PC, non-consuming probiotics groups.

There is a growing need to understand the complex interactions between gut microbiota and the underlying causes of diabetes, and to develop novel treatments and prevention strategies with fewer side effects. Diabetics experience an imbalance in their gut microbiota, and this imbalance is associated with key risk factors for diabetes, such as insulin resistance. Probiotics are crucial for improving gut health, helping control blood sugar levels and improving insulin resistance. Therefore, by combining probiotics with sweet potato powder, we aim to offer new ideas for preventing and improving diabetes as a functional food ingredient. This strain exhibited anti-diabetic effects by enhancing glucose and lipid metabolism and improving intestinal microbiota in a mouse model. Furthermore, when fermented with sweet potatoes, it exhibited improved survival and stability, suggesting its potential as a symbiotic material in the food industry. Furthermore, this study provides insight into the synergistic effect of sweet potato powder as a prebiotic, serving as food for probiotics, promoting the growth of beneficial intestinal bacteria and improving the intestinal environment.

## 5. Conclusions

BST.L-601 has potential as a functional material for preventing and improving T2DM. The strain showed anti-diabetic effects in a mouse model by enhancing glucose and lipid metabolism, including improved gut microbiota. Moreover, the confirmed safety of the strain and the enhanced viability and stability with fermentation, accompanied by sweet potato, support the applicability of the strain as a symbiotic material in the functional food industry.

Further studies, particularly mechanistic investigation of the anti-diabetic effects of BST.L-601 using SP and human clinical tests, will highlight its efficacy for successful industrial applications in a profound way.

## Figures and Tables

**Table 1:** Media composition of seed and main culture for BST.L-601.

Components	Seed Culture (g/L)	Main Culture (g/L)
Sweet potato powder	-	0/20
Glucose	30	40
Casein peptone	12	-
Soy peptone	5	10
Yeast extract	10	15
Tween 80	-	1
Ammonium citrate	1	-
Sodium acetate	1	1
Potassium citrate	-	1
Dipotassium phosphate	1	3
Magnesium sulfate	0.1	0.01
Manganese (II) sulfate	0.1	0.1
Calcium chloride	-	0.5

**Table 2:** Summary of safety assay results from BST.L-601.

Safety Assay	Result (BST.L-601)
Antibiotics susceptibility	Satisfies EFSA standards
Virulence genes	Not detected
Hemolytic activity	Gamma (γ) hemolysis (non-hemolytic)
Gelatin hydrolytic activity	Not observed
Biogenic amine (BA) productivity	Not observed

**Table 3:** Comparison of organ weight.

(g)	NC	PC	601-L	601-H
Spleen	47.2 ± 25.5	32.6 ± 3.0	34.9 ± 6.1	38.9 ± 11.1
Kidney	370.2 ± 21.6	370.6 ± 53.8	375.5 ± 17.1	357.2 ± 59.3
Pancreas	163.5 ± 31.3	115.9 ± 11.3 **	99.0 ± 20.7 ***	113.1 ± 17.6 **
Liver	2049.4 ± 404.8	2457.8 ± 391.4	2014.3 ± 354.6	2233.3 ± 308.5

NC, untreated group; PC, metformin-treated group; 601-L, low concentration of BST.L-601-treated group; 601-H, high concentration of BST.L-601-treated group. Values are the means ± SD (*n* = 5), determined by one-way ANOVA and Dunnett’s multiple comparison test (** *p* < 0.01, *** *p* < 0.001).

**Table 4:** Index changes in serum analysis from 8-week animal treatment.

Serum Index	NC	PC	601-L	601-H
ALP (U/L)	166.60 ± 39.22	141.80 ± 14.31	148.17 ± 21.11	181.60 ± 41.13
GOT (U/L)	296.82 ± 218.24	288.52 ± 35.69	150.02 ± 34.08	242.82 ± 87.94
GPT (U/L)	72.60 ± 25.42	89.80 ± 30.51	59.80 ± 14.41	125.00 ± 39.84 *
BUN (mg/dL)	21.00 ± 1.90	31.18 ± 9.71 *	25.12 ± 5.05	22.84 ± 2.69
CREA (mg/dL)	0.77 ± 0.13	0.74 ± 0.04	0.77 ± 0.06	0.79 ± 0.06
Glucose (mg/dL)	607.40 ± 59.71	600.60 ± 67.69	592.40 ± 21.45 *	489.00 ± 69.44
Triglyceride (mg/dL)	81.40 ± 32.61	88.60 ± 45.99	101.60 ± 43.94	66.00 ± 31.02
Cholesterol (mg/dL)	144.60 ± 23.37	141.00 ± 9.03	111.40 ± 14.19 **	138.20 ± 9.36
HDL (mg/dL)	68.60 ± 4.16	76.00 ± 4.06 *	78.40 ± 2.88 **	73.80 ± 4.76
LDL (mg/dL)	10.20 ± 1.10	11.00 ± 1.79	9.60 ± 0.55	10.60 ± 1.14
LDL/HDL (fold)	1.00 ± 0.06	0.92 ± 0.09	0.83 ± 0.04 **	0.97 ± 0.12

NC, untreated group; PC, metformin-treated group; 601-L, low concentration of BST.L-601-treated group; 601-H, high concentration of BST.L-601-treated group. Values are the means ± SD (*n* = 5), determined by one-way ANOVA and Dunnett’s multiple comparison test (* *p* < 0.05, ** *p* < 0.01).

## Data Availability

The original contributions presented in the study are included in the article/Supplementary Materials. Further inquiries can be directed to the corresponding authors.

## Supplementary Materials

The following supporting information can be downloaded at https://www.mdpi.com/article/10.3390/foods14244230/s1, Table S1. Composition of cryoprotectant for BST.L-601. Table S2. Comparison of antibiotic susceptibility of BST.L-601 and EFSA cut-off values. Table S3. Screening results for virulence genes of BST.L-601 using VirulenceFinder 2.0. Figure S1. Identification of L. rhamnosus BST.L-601 strain based on 16s RNA and NCBI/BLAST software. (A) Analysis of base sequence of BST.L-601 based on 16s RNA. (B) Top 15 identical strains based on BLAST identity score (%). Figure S2. Identification of *L. rhamnosus* BST.L-601 strain based on 16s RNA and NCBI/BLAST software. (A) Analysis of base sequence of BST.L-601 based on 16s RNA. (B) Top 15 identical strains based on BLAST identity score (%). Figure S3. Hemolytic activity assessment of *L. rhamnosus* BST.L-601 and control strains. Each strain was streaked on a blood agar plate and cultivated. (A) *L. rhamnosus* BST.L-601, (B) *L. rhamnosus* GG, (C) *E. coli*. Figure S4. Gelatin hydrolysis assessment of BST.L-601 and control substances. Broth of BST.L-601 and control substances, medium and protease, were inoculated to nutrient gelatin media and incubated. (A) Protease. (B) Medium of BST.L-601. (C) BST.L-601 broth. Figure S5. Biogenic amine production assessment of BST.L-601 and control strain. Each strain was streaked on a Bovid-Cid and Holzapfel medium agar plate containing (A,D) L-tyrosine, (B,E) L-ornithine, (C,F) L-histidine and cultivated. (A–C) *L. rhamnosus* BST.L-601, (D–F) *L. rhamnosus* GG. Figure S6. Bile tolerance assessment of *L. rhamnosus* BST.L-601 and control strain. Each strain was streaked on an MRS agar plate (A,C) and an MRS agar plate containing taurodeolycholic acid (B, D) and cultivated. (A,B) *L. rhamnosus* BST.L-601, (A,B) *L. rhamnosus* GG (C,D). Figure S7. Mucin degradation activity assessment of *L. rhamnosus* BST.L-601 and control strains. Each strain was pipetted on a mucin-containing agar plate and then cultivated and stained with amido-black solution. (A) *L. rhamnosus* BST.L-601, (B) *L. rhamnosus* GG (C) *Bacillus* spp. Figure S8. Caco-2 cell adhesion activity assessment of BST.L-601 and control strain. Caco-2 colorectal cells were treated with diluted broth of each strain and CFU of the broth before (A,C) and after (B,D) treatment were compared using an MRS agar plate. (A,B) *L. rhamnosus* BST.L-601, (C,D) *L. rhamnosus* GG. Figure S9. Changes in body weight, water intake, fasting blood glucose, and OGTT of mice fed different diets. (A) Body weight. (B) Water intake volume. (C) Fasting blood glucose. (D) OGTT. Mice were given restricted diets with the stated doses of metformin and BST.L-601. Body weight and water intake volume were measured weekly, and fasting blood glucose was measured biweekly. NC, untreated group; PC, metformin-treated group; 601-L, low concentration of BST.L-601-treated group; 601-H, high concentration of BST.L-601-treated group. Values are the means ± SD (*n* = 5).

## Abbreviations

The following abbreviations are used in this manuscript:

2-NBDG	2-deoxy-2-[(7-nitro-2,1,3-benzoxadiazol-4-yl) amino]-D-glucose
EFSA	European Food Safety Authority
FBG	Fasting blood glucose
FMT	Fecal microbiota transplantation
GLUT4	Glucose transporter type 4
H&E staining	Hematoxylin and eosin staining
IHC	Immunohistochemistry
IRS-1	Insulin receptor substrate 1
MIC	Minimal inhibitory concentration
OGTT	Oral glucose tolerance test
PCoA	Principal Coordinates Analysis
