# Supplementary material for: Evaluation of Potential Anti-Diabetic Synbiotic Formulation of Lacticaseibacillus rhamnosus BST.L-601 Using db/db Mice
Source: Foods. 2025 Dec 9;14(24):4230. doi: 10.3390/foods14244230 (PMC12731962; doi:10.3390/foods14244230)
Supplement: Supplementary file 1 [file foods-14-04230-s001.zip › foods-3975195-supplementary.pptx]

## Slide 1
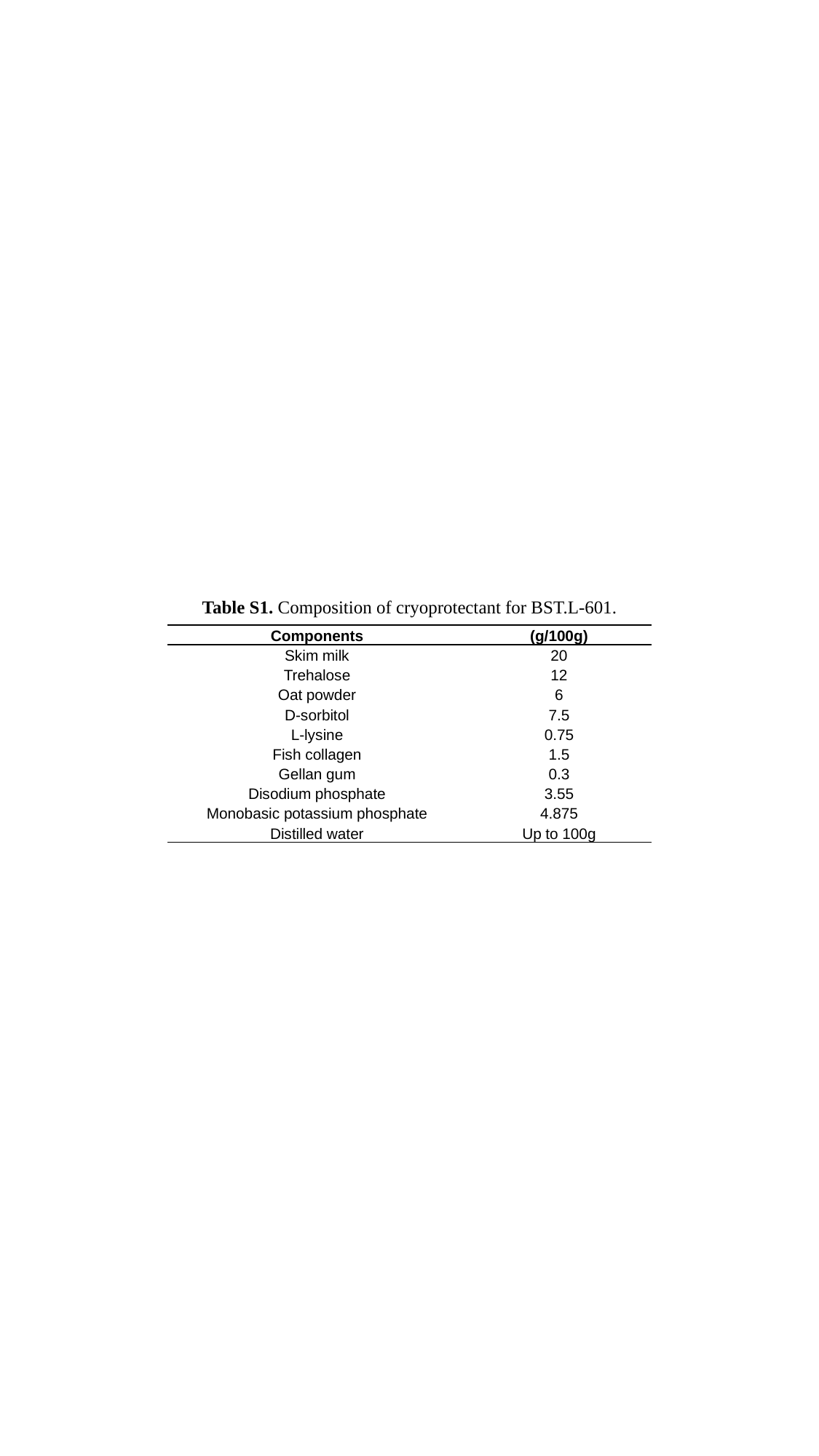

Table S1. Composition of cryoprotectant for BST.L-601.
| Components | (g/100g) |
| --- | --- |
| Skim milk | 20 |
| Trehalose | 12 |
| Oat powder | 6 |
| D-sorbitol | 7.5 |
| L-lysine | 0.75 |
| Fish collagen | 1.5 |
| Gellan gum | 0.3 |
| Disodium phosphate | 3.55 |
| Monobasic potassium phosphate | 4.875 |
| Distilled water | Up to 100g |

## Slide 2
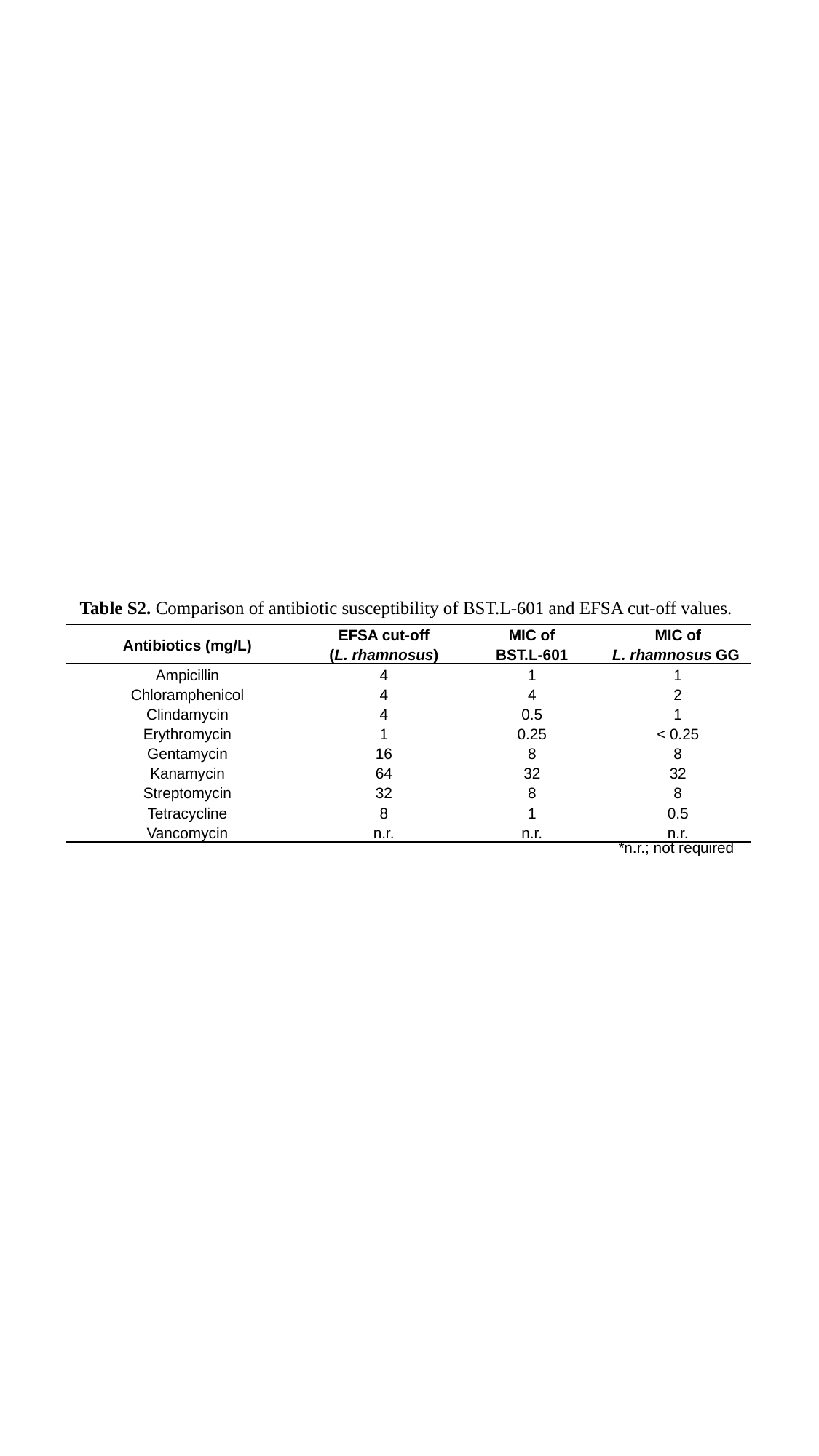

Table S2. Comparison of antibiotic susceptibility of BST.L-601 and EFSA cut-off values.
| Antibiotics (mg/L) | EFSA cut-off (L. rhamnosus) | MIC of BST.L-601 | MIC of L. rhamnosus GG |
| --- | --- | --- | --- |
| Ampicillin | 4 | 1 | 1 |
| Chloramphenicol | 4 | 4 | 2 |
| Clindamycin | 4 | 0.5 | 1 |
| Erythromycin | 1 | 0.25 | < 0.25 |
| Gentamycin | 16 | 8 | 8 |
| Kanamycin | 64 | 32 | 32 |
| Streptomycin | 32 | 8 | 8 |
| Tetracycline | 8 | 1 | 0.5 |
| Vancomycin | n.r. | n.r. | n.r. |
*n.r.; not required

## Slide 3
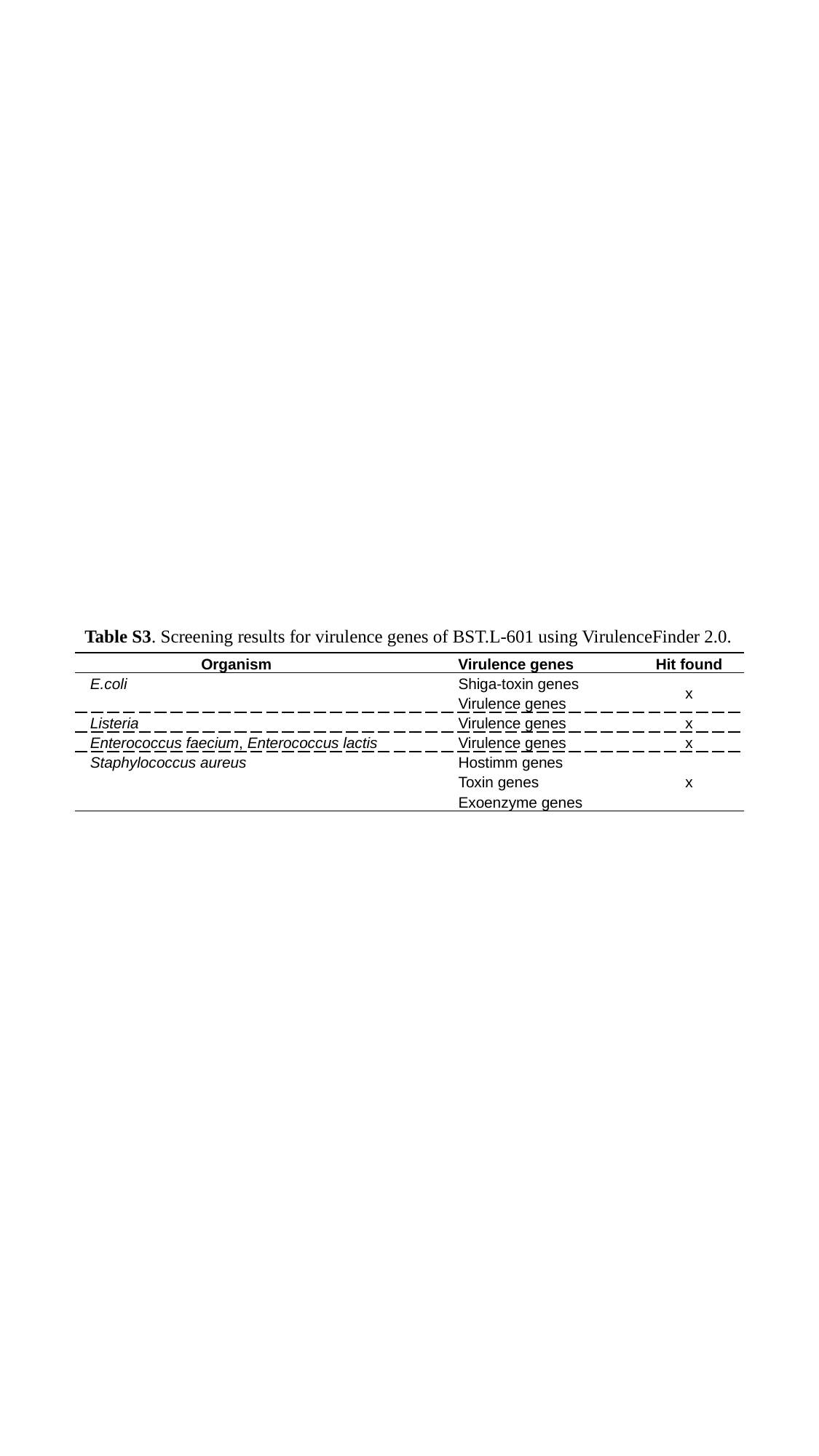

Table S3. Screening results for virulence genes of BST.L-601 using VirulenceFinder 2.0.
| Organism | Virulence genes | Hit found |
| --- | --- | --- |
| E.coli | Shiga-toxin genes | x |
| | Virulence genes | |
| Listeria | Virulence genes | x |
| Enterococcus faecium, Enterococcus lactis | Virulence genes | x |
| Staphylococcus aureus | Hostimm genes | x |
| | Toxin genes | |
| | Exoenzyme genes | |

## Slide 4
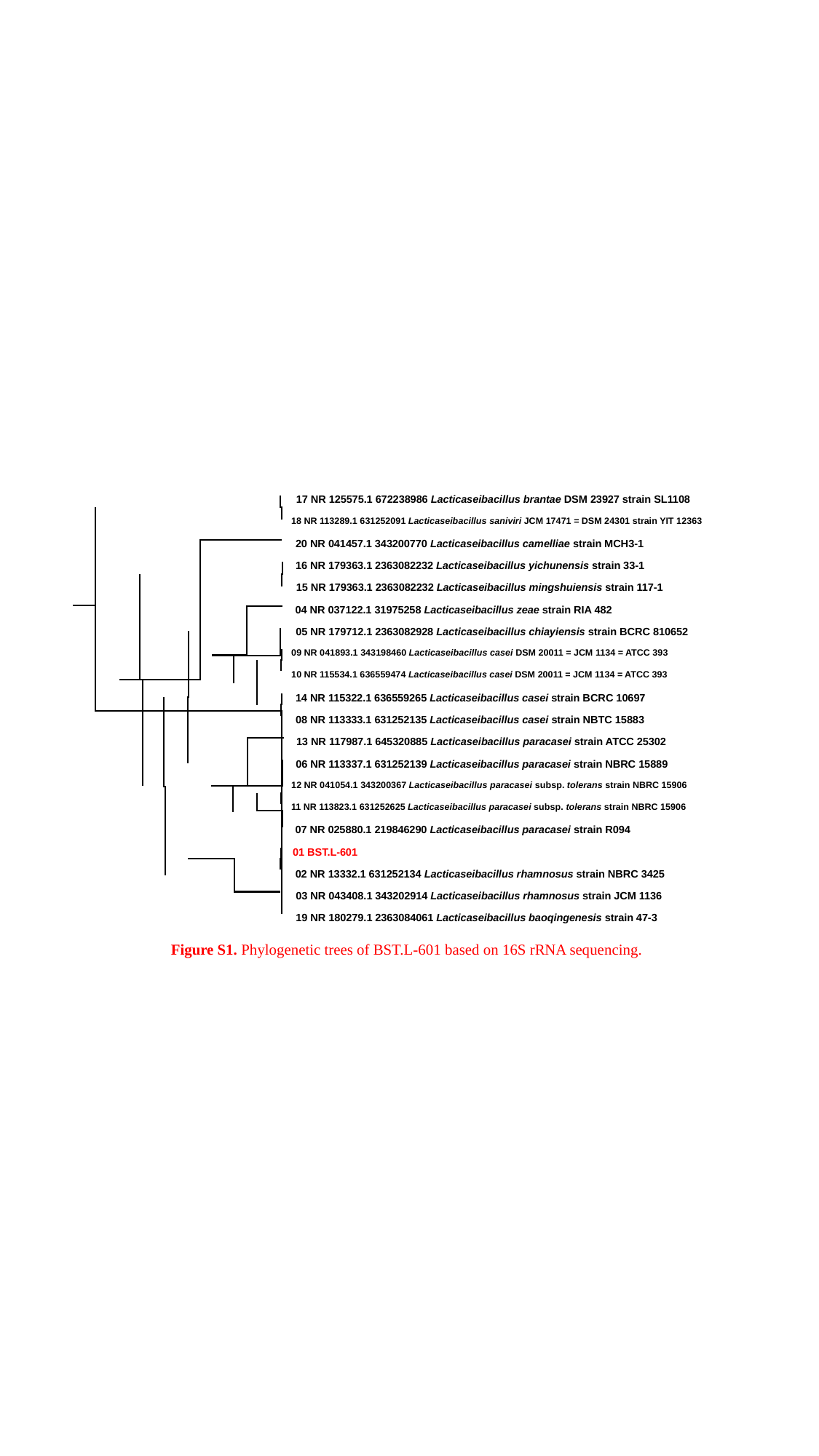

17 NR 125575.1 672238986 Lacticaseibacillus brantae DSM 23927 strain SL1108
18 NR 113289.1 631252091 Lacticaseibacillus saniviri JCM 17471 = DSM 24301 strain YIT 12363
20 NR 041457.1 343200770 Lacticaseibacillus camelliae strain MCH3-1
16 NR 179363.1 2363082232 Lacticaseibacillus yichunensis strain 33-1
15 NR 179363.1 2363082232 Lacticaseibacillus mingshuiensis strain 117-1
04 NR 037122.1 31975258 Lacticaseibacillus zeae strain RIA 482
05 NR 179712.1 2363082928 Lacticaseibacillus chiayiensis strain BCRC 810652
09 NR 041893.1 343198460 Lacticaseibacillus casei DSM 20011 = JCM 1134 = ATCC 393
10 NR 115534.1 636559474 Lacticaseibacillus casei DSM 20011 = JCM 1134 = ATCC 393
14 NR 115322.1 636559265 Lacticaseibacillus casei strain BCRC 10697
08 NR 113333.1 631252135 Lacticaseibacillus casei strain NBTC 15883
13 NR 117987.1 645320885 Lacticaseibacillus paracasei strain ATCC 25302
06 NR 113337.1 631252139 Lacticaseibacillus paracasei strain NBRC 15889
12 NR 041054.1 343200367 Lacticaseibacillus paracasei subsp. tolerans strain NBRC 15906
11 NR 113823.1 631252625 Lacticaseibacillus paracasei subsp. tolerans strain NBRC 15906
07 NR 025880.1 219846290 Lacticaseibacillus paracasei strain R094
01 BST.L-601
02 NR 13332.1 631252134 Lacticaseibacillus rhamnosus strain NBRC 3425
03 NR 043408.1 343202914 Lacticaseibacillus rhamnosus strain JCM 1136
19 NR 180279.1 2363084061 Lacticaseibacillus baoqingenesis strain 47-3
Figure S1. Phylogenetic trees of BST.L-601 based on 16S rRNA sequencing.

## Slide 5
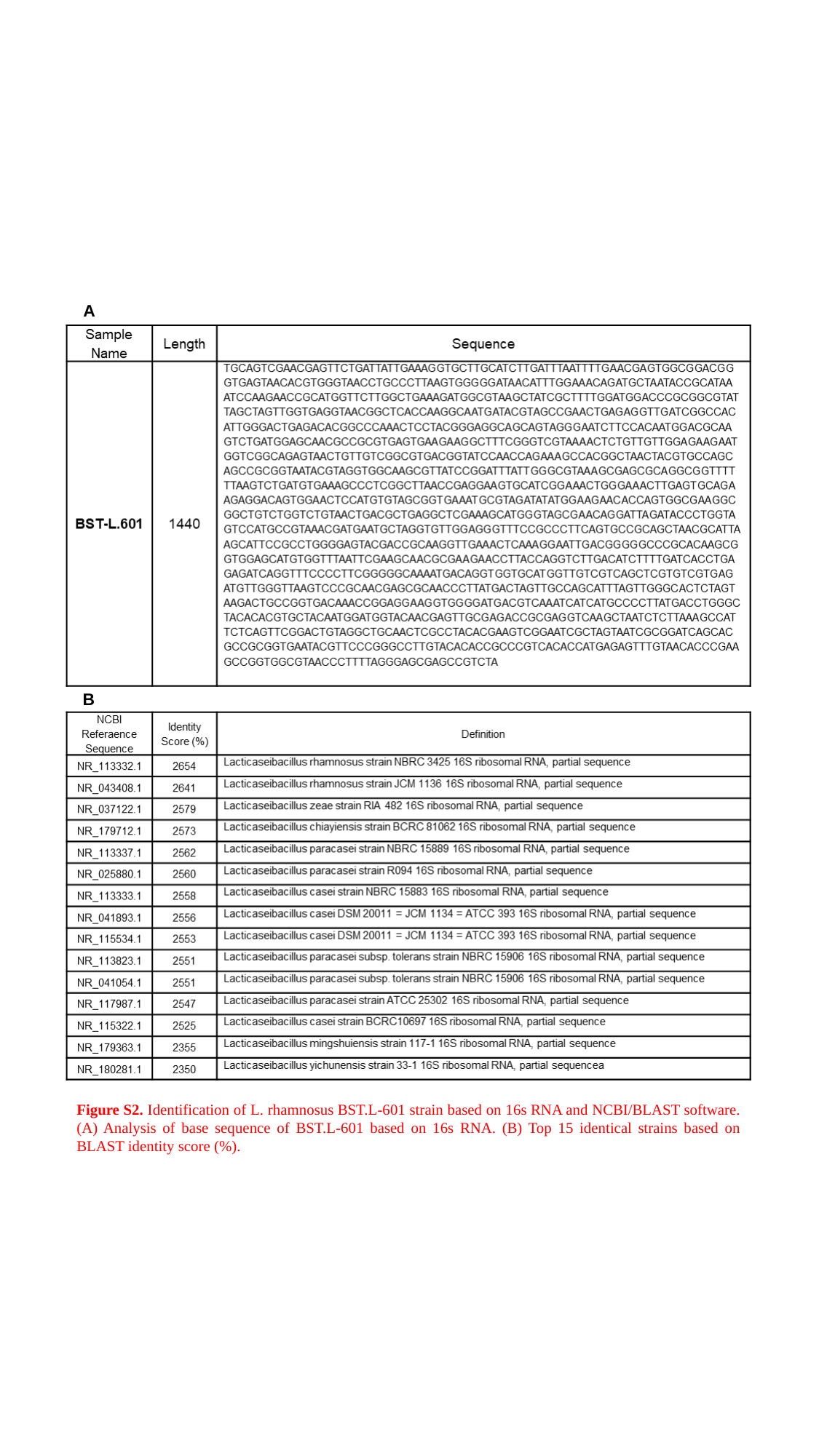

A
B
Figure S2. Identification of L. rhamnosus BST.L-601 strain based on 16s RNA and NCBI/BLAST software. (A) Analysis of base sequence of BST.L-601 based on 16s RNA. (B) Top 15 identical strains based on BLAST identity score (%).

## Slide 6
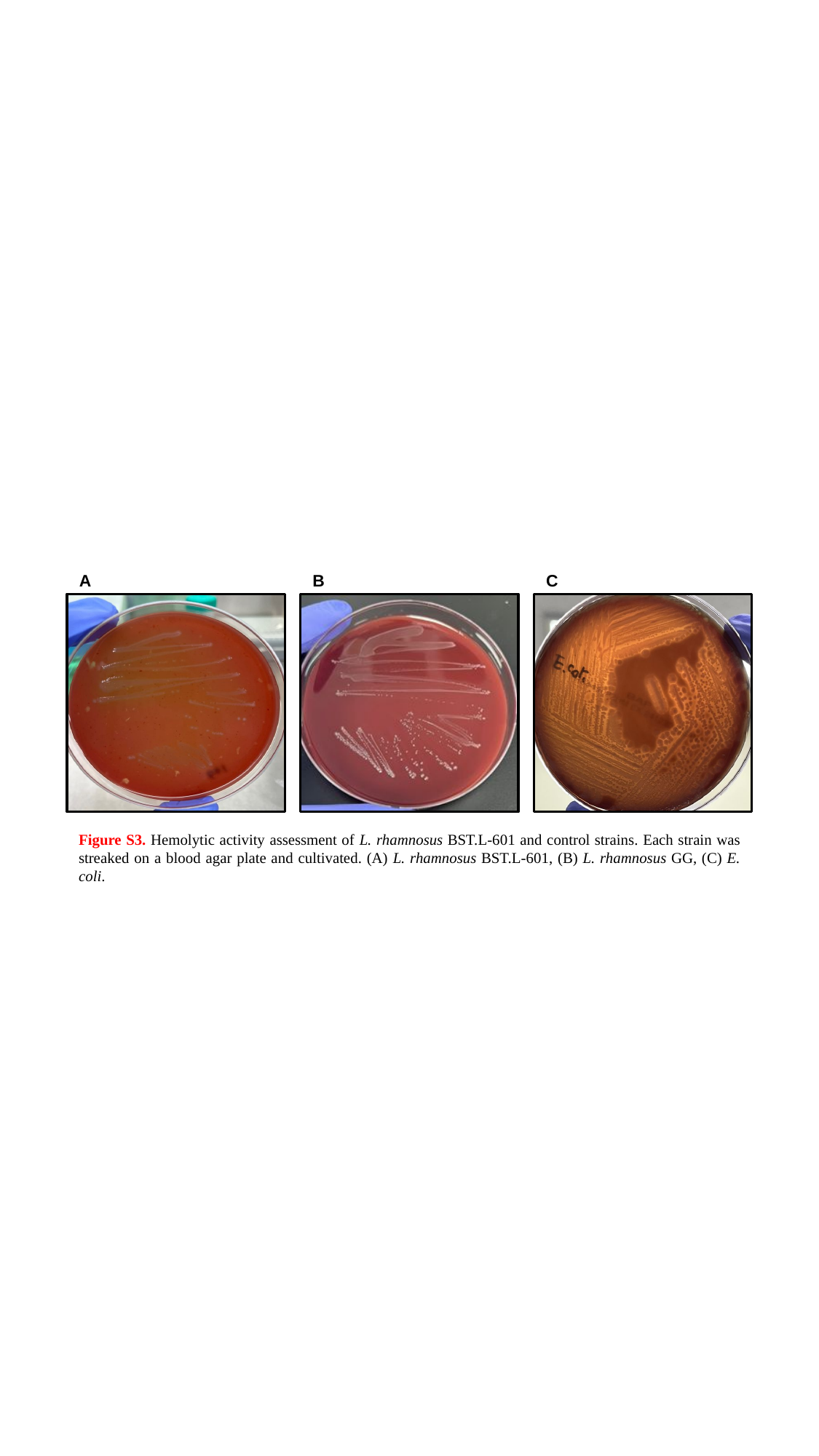

A
B
C
Figure S3. Hemolytic activity assessment of L. rhamnosus BST.L-601 and control strains. Each strain was streaked on a blood agar plate and cultivated. (A) L. rhamnosus BST.L-601, (B) L. rhamnosus GG, (C) E. coli.

## Slide 7
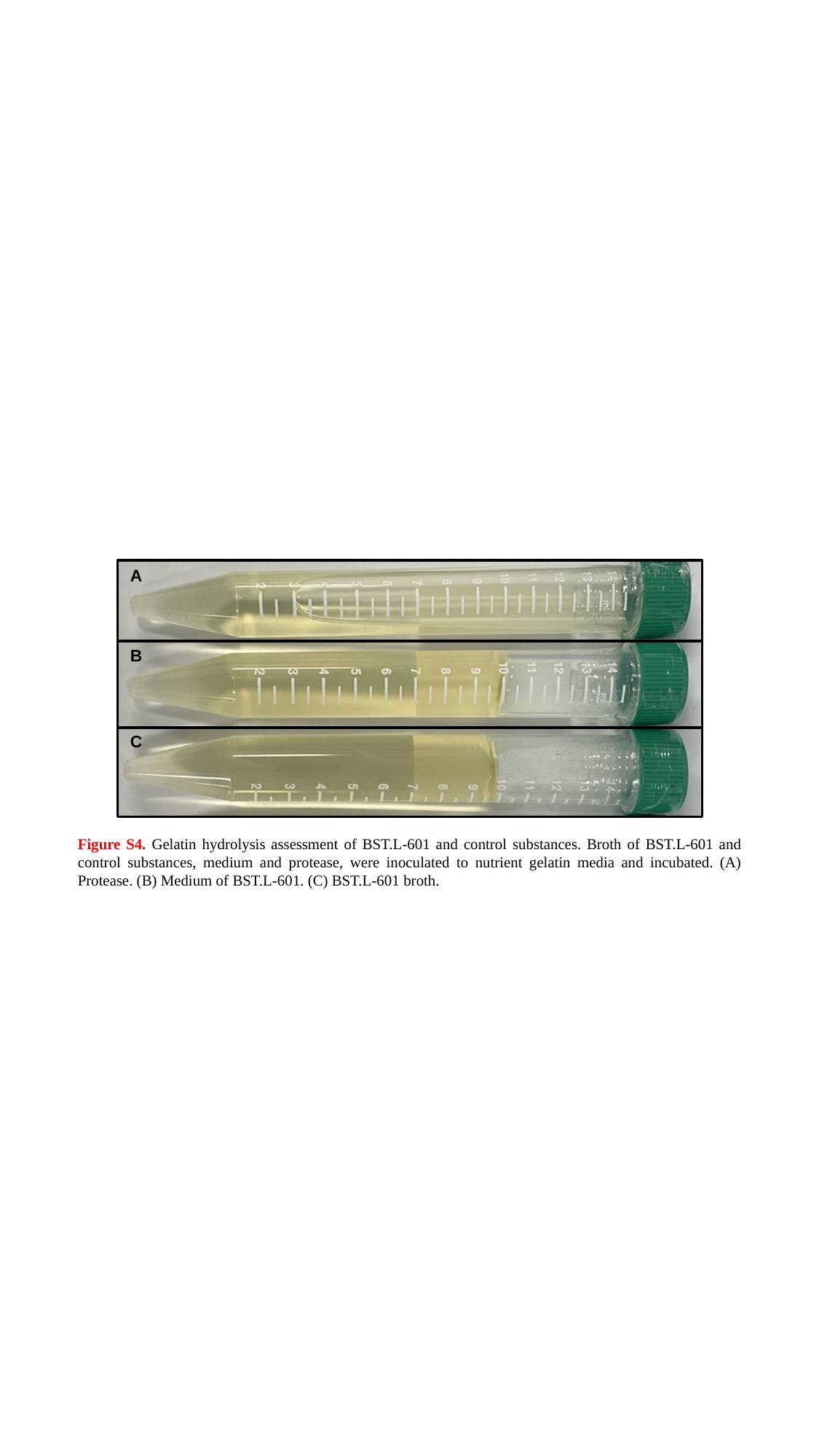

A
B
C
Figure S4. Gelatin hydrolysis assessment of BST.L-601 and control substances. Broth of BST.L-601 and control substances, medium and protease, were inoculated to nutrient gelatin media and incubated. (A) Protease. (B) Medium of BST.L-601. (C) BST.L-601 broth.

## Slide 8
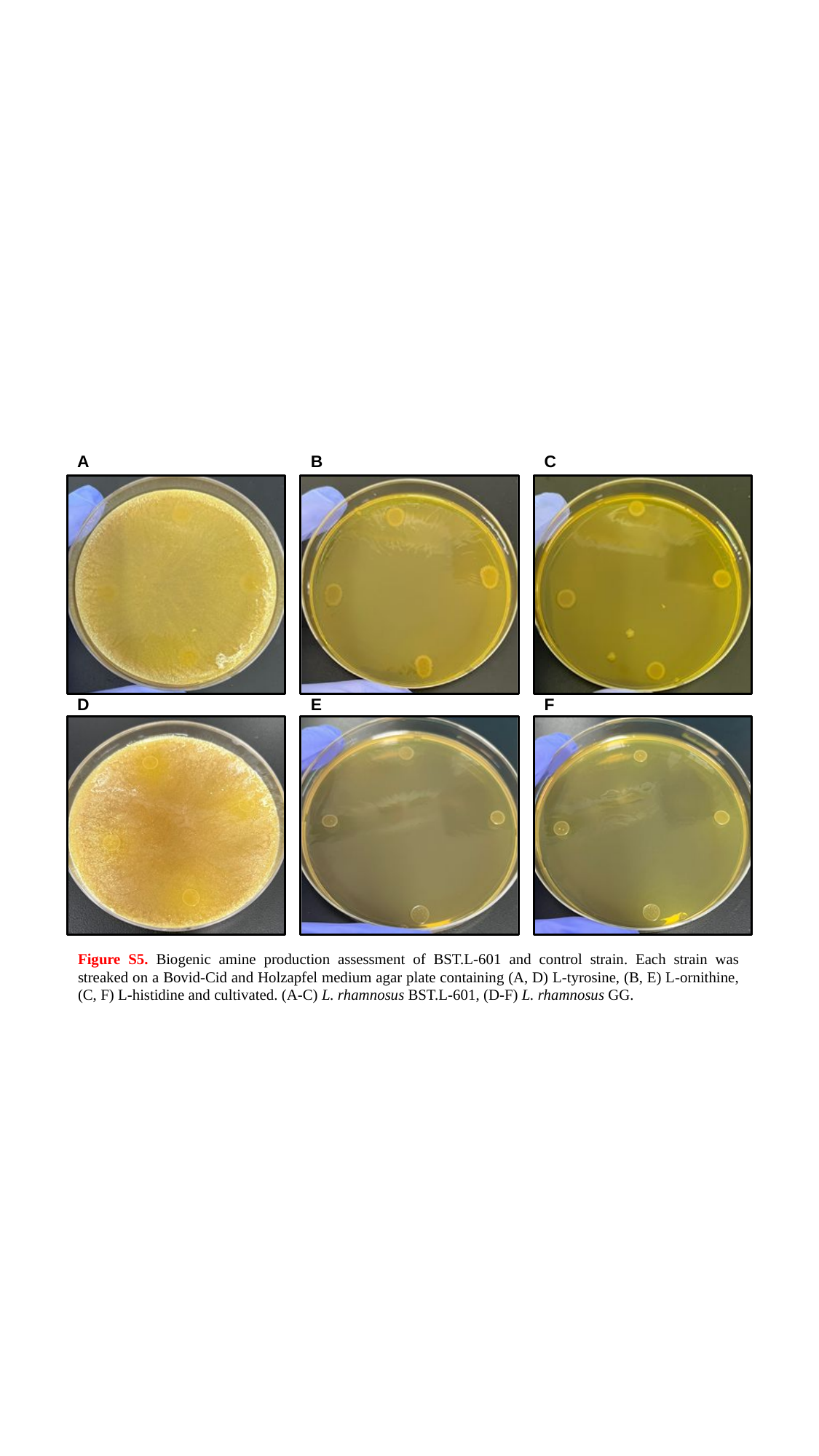

A
B
C
D
E
F
Figure S5. Biogenic amine production assessment of BST.L-601 and control strain. Each strain was streaked on a Bovid-Cid and Holzapfel medium agar plate containing (A, D) L-tyrosine, (B, E) L-ornithine, (C, F) L-histidine and cultivated. (A-C) L. rhamnosus BST.L-601, (D-F) L. rhamnosus GG.

## Slide 9
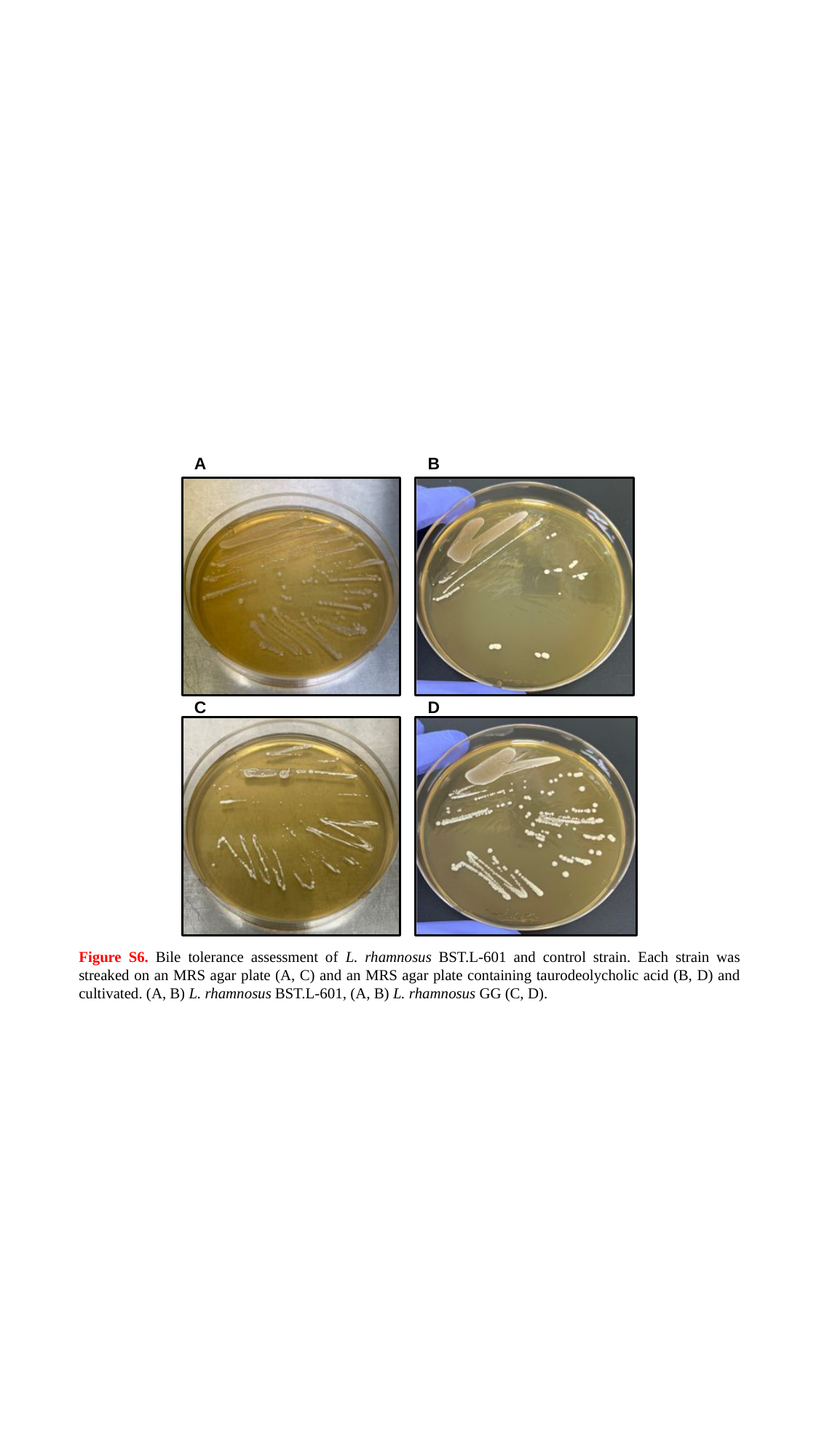

A
B
C
D
Figure S6. Bile tolerance assessment of L. rhamnosus BST.L-601 and control strain. Each strain was streaked on an MRS agar plate (A, C) and an MRS agar plate containing taurodeolycholic acid (B, D) and cultivated. (A, B) L. rhamnosus BST.L-601, (A, B) L. rhamnosus GG (C, D).

## Slide 10
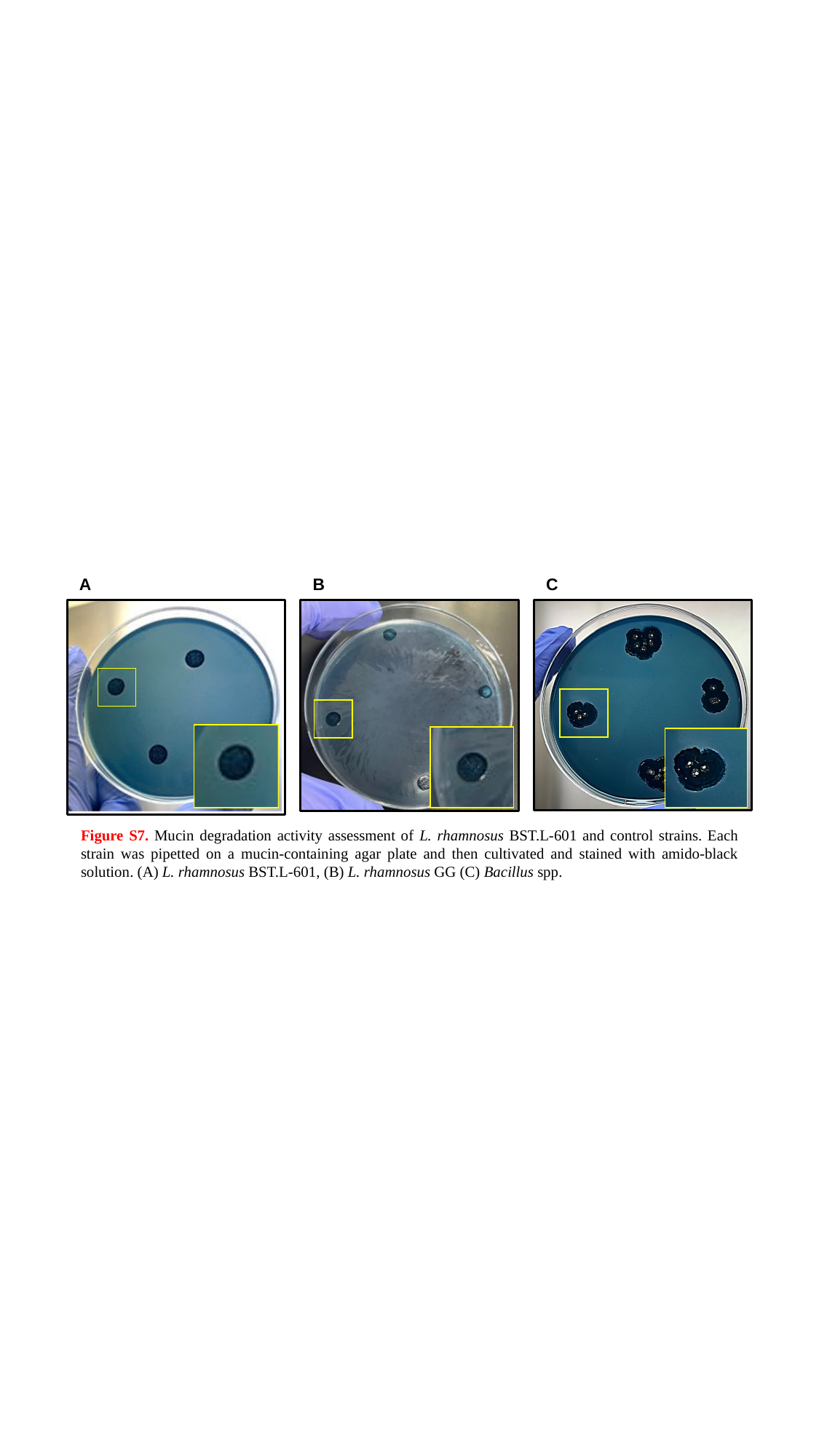

A
B
C
Figure S7. Mucin degradation activity assessment of L. rhamnosus BST.L-601 and control strains. Each strain was pipetted on a mucin-containing agar plate and then cultivated and stained with amido-black solution. (A) L. rhamnosus BST.L-601, (B) L. rhamnosus GG (C) Bacillus spp.

## Slide 11
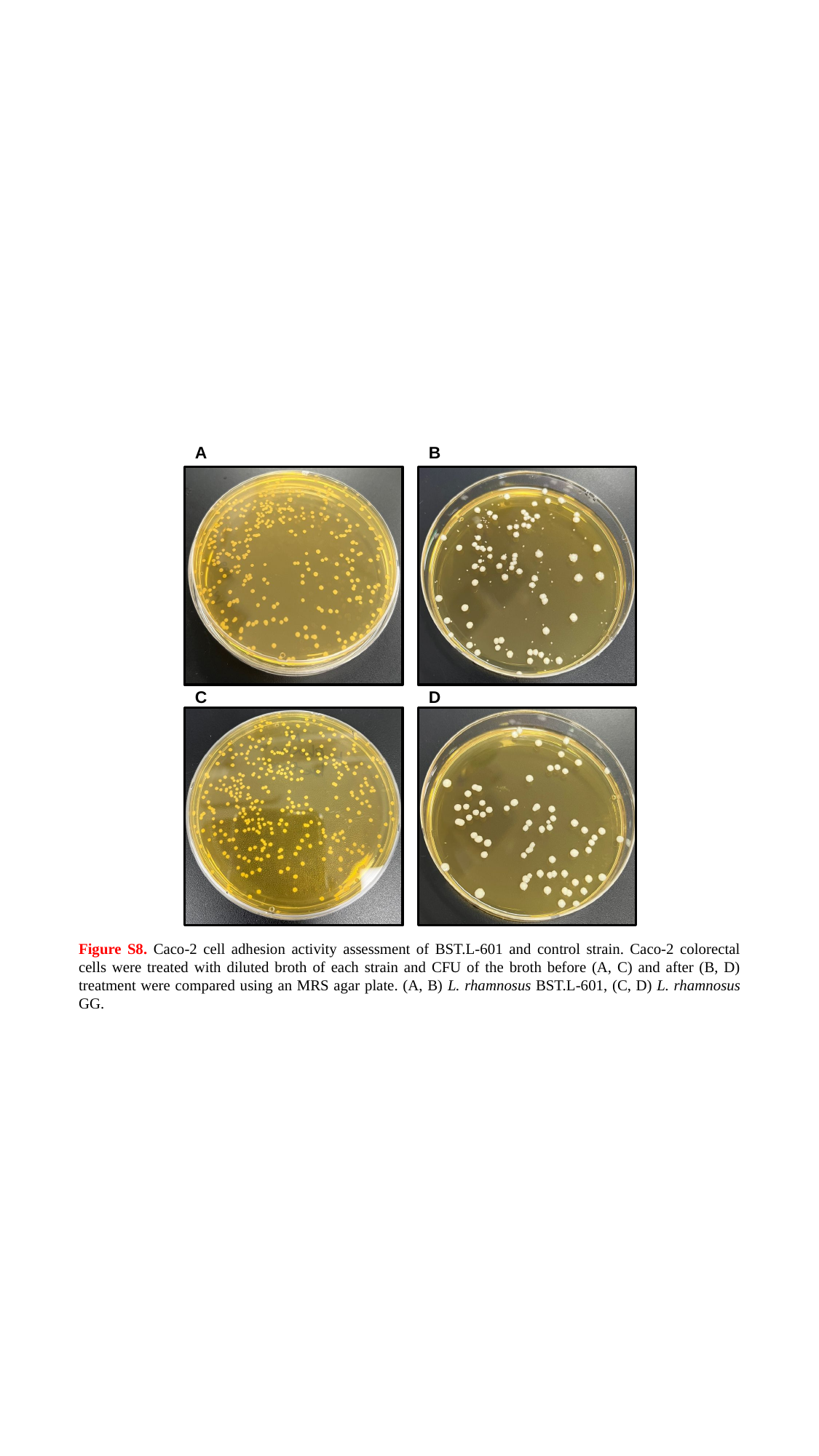

A
B
C
D
Figure S8. Caco-2 cell adhesion activity assessment of BST.L-601 and control strain. Caco-2 colorectal cells were treated with diluted broth of each strain and CFU of the broth before (A, C) and after (B, D) treatment were compared using an MRS agar plate. (A, B) L. rhamnosus BST.L-601, (C, D) L. rhamnosus GG.

## Slide 12
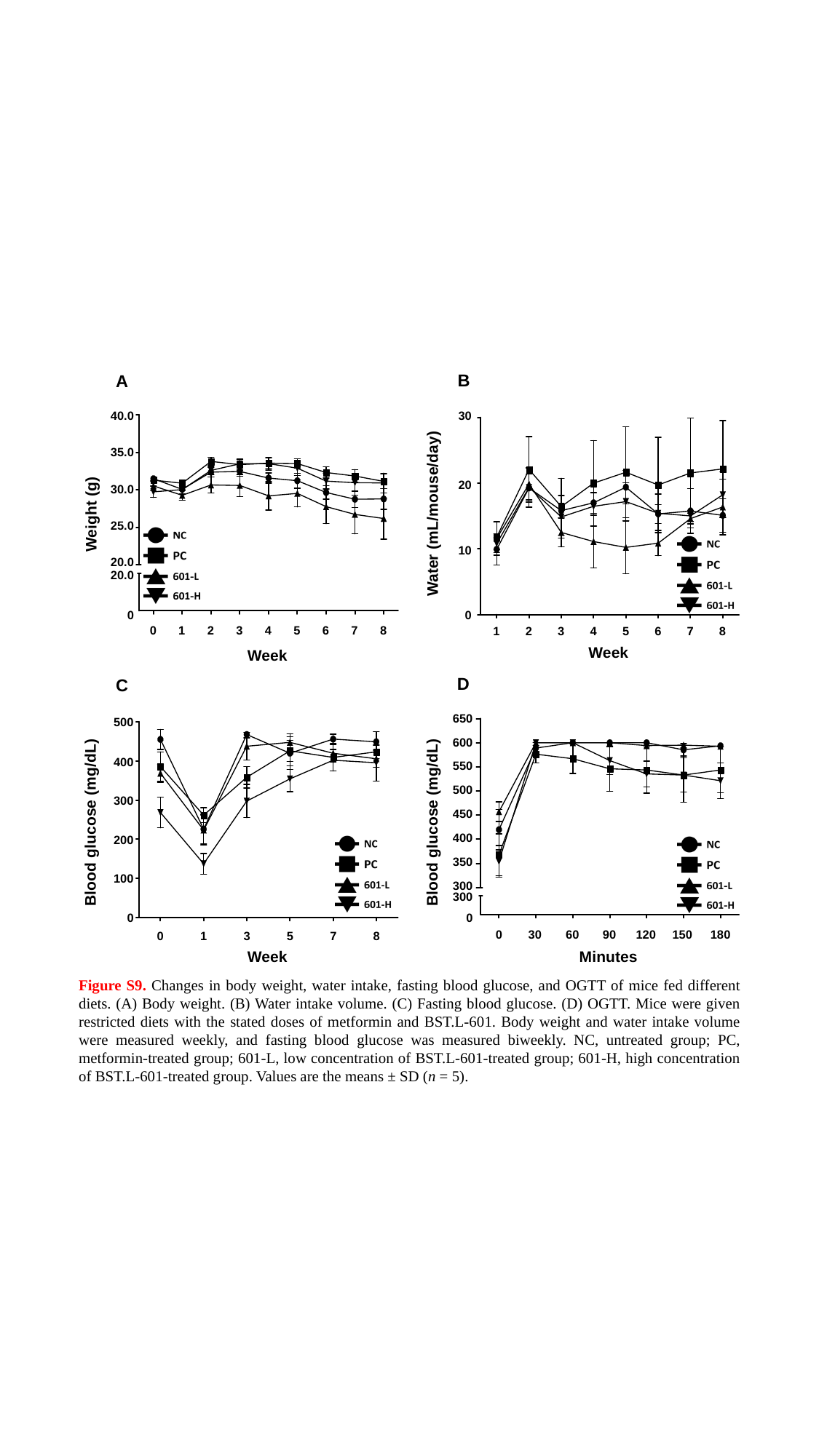

B
A
40.0
30
35.0
20
30.0
Water (mL/mouse/day)
Weight (g)
25.0
10
20.0
20.0
0
0
0
1
2
3
4
5
6
7
8
1
2
3
4
5
6
7
8
Week
Week
D
C
650
500
600
400
550
500
300
450
Blood glucose (mg/dL)
Blood glucose (mg/dL)
400
200
350
100
300
300
0
0
0
30
60
90
150
180
120
0
1
3
5
7
8
Week
Minutes
Figure S9. Changes in body weight, water intake, fasting blood glucose, and OGTT of mice fed different diets. (A) Body weight. (B) Water intake volume. (C) Fasting blood glucose. (D) OGTT. Mice were given restricted diets with the stated doses of metformin and BST.L-601. Body weight and water intake volume were measured weekly, and fasting blood glucose was measured biweekly. NC, untreated group; PC, metformin-treated group; 601-L, low concentration of BST.L-601-treated group; 601-H, high concentration of BST.L-601-treated group. Values are the means ± SD (n = 5).
